# Mycosporine-Like Amino Acids and Their Derivatives as Natural Antioxidants

**DOI:** 10.3390/antiox4030603

**Published:** 2015-09-07

**Authors:** Naoki Wada, Toshio Sakamoto, Seiichi Matsugo

**Affiliations:** School of Natural System, College of Science and Engineering, Kanazawa University, Kakuma, Kanazawa 920-1192, Japan; E-Mails: naoki-wada@se.kanazawa-u.ac.jp (N.W.); tsakamot@staff.kanazawa-u.ac.jp (T.S.)

**Keywords:** mycosporine-like amino acids, pigment, sun screening, radical scavenging, antioxidant, glycosylation, anhydrobiosis

## Abstract

Mycosporine-like amino acids (MAAs) are water-soluble molecules that absorb UV-A and UV-B radiation and disperse the energy as heat. MAAs show great diversity in their molecular structures, which exhibit a range of molecular weights spanning 188 to 1050 Daltons. MAAs are utilized in a wide variety of organisms including prokaryotes and eukaryotic micro-organisms that inhabit aquatic, terrestrial, and marine environments. These features suggest that MAAs are stable and fundamental molecules that allow these organisms to live under UV irradiation. MAAs are thought to have been greatly important to ancient forms of life on Earth, functioning as a primary sunscreen to reduce short-wavelength light. Structurally different MAAs might have been developed in MAA-producing organisms during their environmental adaptation. Harmful irradiation directly damages biomolecules, including lipids, proteins and DNA, and induces oxidative stress through radical-propagating processes. Thus, MAAs are expected to play an additional role in the antioxidant system. This review focuses on MAAs with radical scavenging activities. To cover all the reported MAAs known thus far, we surveyed the CAS database and have summarized the structures and the chemical and physical properties of these MAAs, including their antioxidant activities.

## 1. Introduction

Photoautotrophs oxidize water to form oxygen molecules via the photochemical reaction of photosynthesis, while aerobes utilize oxygen molecules as electron acceptors in respiratory electron transport. Both types of organisms, with their respective energy acquisition mechanisms, are confronted with oxidative stress. Oxidative stress causes unavoidable problems in growing and living on the Earth. In the process of photosynthesis, as well as in respiration, reactive oxygen species (ROS) are produced, and ROS damage biomolecules, including lipids, protein and DNA. Thus, organisms must have antioxidant systems to protect their bodies from long-lived and short-lived ROS. First, the suppression of ROS formation is important; sunscreening is one suppression mechanism. Second, direct scavenging of ROS by antioxidants and antioxidant enzymes occurs. Third, the reacted antioxidants (inactive forms) can be regenerated by other antioxidants to enable further scavenging in a process known as the antioxidant network.

Natural antioxidants are low-molecular-weight substances that can effectively suppress the generation of ROS and/or prevent oxidative damage to biomolecules. Natural antioxidants are classified into five categories: organosulfur compounds, flavonoids, phenolic compounds, carotenoids, and vitamins. This variety of antioxidant species has evolved throughout the environmental adaptation of organisms; for example, land plants commonly contain vitamin C, which is thought to function in an antioxidant that is required for adaptation to terrestrial environments. Herbivores obtain vitamin C in their diets and utilize it in their own antioxidant systems; thus, vitamin C functions in diverse organisms as a general antioxidant. Moreover, there are unique antioxidants produced as secondary metabolites; e.g., anthocyanin synthesized in specific tissue of a certain taxonomic group of plants and accumulate under specific and/or stress conditions. Various anthocyanin derivatives bearing various carbohydrate moieties are well known in plants, which are thought to have developed as an adaptation to biotic and/or abiotic environments. Thus, there are general and specific natural antioxidants that are selected and combined to develop unique antioxidant systems in organisms according to a particular environmental adaptation strategy.

**Figure 1:**
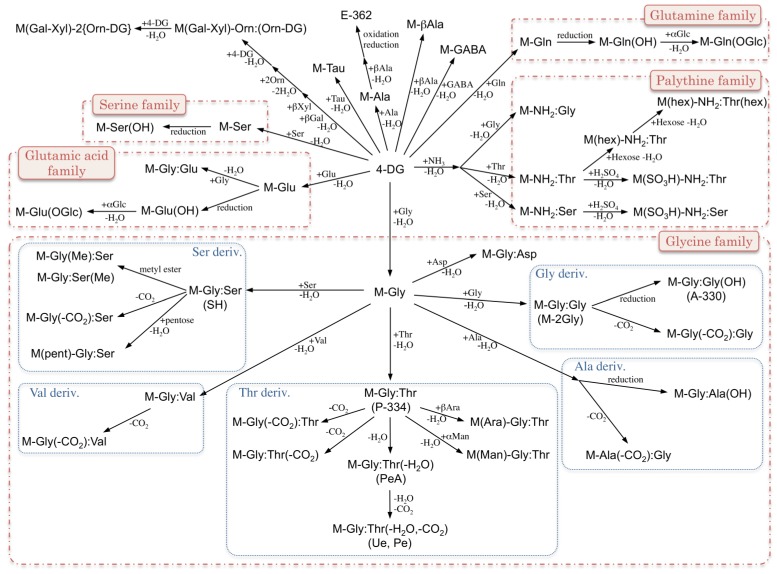
Structural relationships of various mycosporine-like amino acids (MAAs). Note that ammonia is not used for MAA biosynthesis due to its toxicity; however, it is useful to simplify the figure by explaining the structural relationships of various types of MAAs.

Mycosporine-like amino acids (MAAs) are water-soluble molecules that absorb UV-A and -B light. Their absorption maxima appear between 268 and 362 nm depending on their molecular structure. These UV-absorbing MAA compounds are widely distributed in nature, typically in organisms that are exposed to high-intensity light, such as cyanobacteria and other prokaryotes, eukaryotic micro-organisms (e.g., microalgae and fungi), marine macroalgae (both green and red algae), corals, terrestrial lichens; and other marine organisms that accumulate MAAs from their feed [1]. The photo-physical and photo-chemical properties (e.g., UV-VIS absorption spectrum and photostability in the excited state) of MAAs must be uniquely adapted to the stressful habitats of MAA-producing organisms under strong irradiation. MAAs can be synthesized through the conjugation of amino acids to an intermediate produced in a common metabolic pathway, and the resultant MAA structural core is so primitive that the MAAs are considered to be potential molecular pioneers among natural sunscreens. This feature may be related to the distribution of MAAs, which are often found in marine organisms. The substitution of different amino acids into the MAA core structure can produce a variety of MAA species. Variation in their chemical structures is one of the characteristic features of MAA molecular species and offers a great advantage to MAA-producing organisms by facilitating adaptation to harsh environments with natural sunscreen and antioxidant functions. The original MAA producer is thought to be a cyanobacterium, and the genes involved in MAA biosynthesis likely transferred to the other organisms. Two biosynthesis pathways have been proposed: (1) MAAs are proposed to be biosynthesized via the shikimate pathway [2], which is known for the synthesis of aromatic amino acids. The precursor of the six-membered carbon ring common to all MAAs is 3-dehydroquinate (3-DHQ). 3-DHQ transforms into gadusol and then 4-deoxygadusol. (2) The other biosynthesis route that has been proposed for the synthesis of 4-deoxygadusol involves sedoheptulose 7-phosphate, which is derived from the pentose phosphate pathway and is converted to 4-deoxygadusol [3]. In both pathways, 4-deoxygadusol is the common precursor used to produce all MAAs and the conjugation with a glycine molecule produces a simple mono-substituted cyclohexenone-type MAA called mycosporine-glycine, which is a common intermediate in the production of di-substituted (aminocyclohexene imine-type) MAAs such as porphyra-334 (P-334) and shinorine (SH) [4]. Although some MAA species could not be simply produced via amino acid substitution to 4-deoxygadusol, most of these species are thought to be synthesized via modification of amino acid side-chains by condensation (for esterification and amidation), dehydration (for double bond formation), decarboxylation (for chain shortening), and oxidation and reduction (for hydroxylation). Further substitution reactions (e.g., sulfonation and glycosylation) are known to occur (Figure 1). Thus, MAAs can be divided into aminocyclohexenone-type or aminocyclohexene imine-type according to the central substructure; amino acid substituents and their derivatives can be further characterized as MAA sulfates and glycosides, as described later.

MAAs thought to primarily act as sunscreen agents and more recently as novel antioxidants. Numerous studies of MAAs from various organisms that are exposed to a high intensity of photosynthetically active radiation (PAR) and harmful ultraviolet radiation (UVR) suggest that MAA accumulation functions mainly as sunscreen in marine organisms. In this context, a number of review articles [5,6,7] have been published. UVR causes pigment degradation, singlet oxygen generation by photo-sensitizer (under relatively longer wavelengths), and radical production by cleavage of chemical bonding (under shorter wavelengths), leading to a cascade of radical reactions. Thus, UVR thought to be one of the strongest sources of oxidative stress, and it is naturally accepted that MAA species also have antioxidative roles. Recently MAAs have been found to play multiple roles against several environmental stresses, because MAA synthesis can be affected by salt (osmotic) [8,9,10,11,12,13], desiccation [14,15] and thermal stresses [7,16]. These environmental factors often enhance oxidative stress and antioxidant properties of MAAs are relevant to the functions *in vivo*.

In this review, we focus on the molecular diversity of the MAA species and summarize antioxidant activities to understand the structure-activity relationship. The antioxidant activities of MAAs are explained in this review; oxygen radical absorbance capacity (ORAC) values, lipid peroxidation inhibition, 2,2-diphenyl-1-picryhydrazyl (DPPH) and 2,2′-azino-bis(3-ethylbenzothiazoline-6-sulphonic acid) (ABTS) radical quenching, superoxide anion radical quenching, singlet oxygen quenching, hydrogen peroxide quenching activities, and physiological activities are summarized in Table A1.

To include the structure and chemical and physical properties of all the reported MAAs, we surveyed the chemical abstract service (CAS) database using the structure similarity search engine in SciFinder. The references for the structure determination of all MAA species described in this review are summarized in Table A2. The antioxidant activities of various MAAs and the physiological activities of some MAAs are reviewed. The contents in this review are divided into two main sections, namely non-derivatized (Section 2, Section 3 and Section 4) and derivatized MAAs (Section 5). The former can be considered the aglycone of the pigment and the latter can be considered the second and/or third MAA-related metabolite that evolved to allow an organism to adapt to a specific environment.

## 2. MAA Precursors (Figure 2)

**Figure 2:**
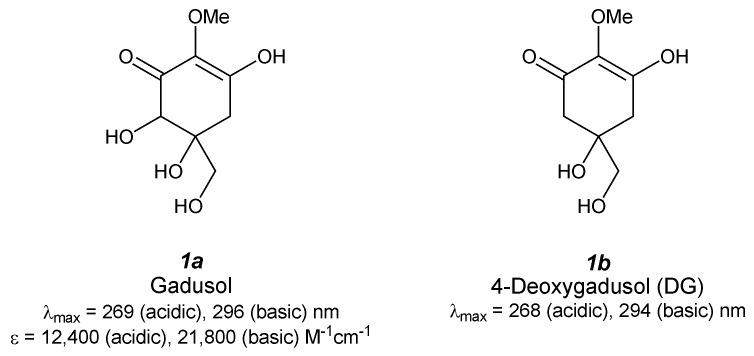
Chemical structure and spectroscopic properties of MAA precursors. Abbreviation is in parenthesis.

### 2.1. Gadusol (Figure 2(1a))

Gadusol was first isolated from ripe eggs of *Scomber scombrus*, *Auxis thazard*, *Belone belone*, *Box boops*, *Trachurus trachurus* [17] and *Gadus morhua* [18] and then was found in various fish roes, including those of haddock, common dab, long rough dab, plaice, and flounder, at a level of approximately 4 mg/g dry wt [19]. Absorption maxima appear at 269 nm (ε = 12,400 M^−1^·cm^−1^) and 296 nm (ε = 21,800 M^−1^·cm^−1^) in acidic and basic media, respectively. ^1^H- and ^13^C-NMR spectra revealed a typical backbone structure of an enolated cyclohexane-1,3-dione, though the stereostructure of gadusol is still unknown. Recently, electrochemical properties were determined by cyclic- and square-wave voltammetry [20]. Peak potentials of the enolic and enolate anion forms of gadusol were 710 ± 5 and 601 ± 9 mV (*vs.* Ag/AgCl), respectively, and the oxidation was irreversible. This result indicates that gadusol has good antioxidant properties and moderate reducing power.

Antioxidant capacity of gadusol has been given much attention recently. The relative oxygen radical absorbance capacity (ORAC) value of gadusol was estimated by comparing the fluorescence decay of fluorescein in the presence of Trolox as a control [21]. The time profile of fluorescence decay shows a lag phase that indicates gadusol is reacting with a peroxy radical much more rapidly than fluorescein. The relative ORAC value of gadusol was determined to be 2.6, which is approximately six-fold larger than that of ascorbic acid (0.46 ± 0.06) and smaller than that of quercetin (4.43 ± 0.21) and rutin (4.03 ± 0.46), that are strong flavonoid antioxidants [22]. The same authors investigated the scavenging reaction of gadusol with a water-soluble stable ABTS radical. The reaction time profile can be separated into two parts: in the first several minutes the reaction occurs rapidly and in the later stage the reaction occurs slowly. This reaction profile is completely different from that of Trolox, which completes the reaction in less than 10 s. Scavenging activity (Trolox equivalent value) of gadusol is almost comparable to that of Trolox (in one min), but after 1 min it becomes larger than that of Trolox (1.27 in 4 min, 1.40 in 6 min). The absorption spectra of gadusol and the gadusolate anion overlap in the UV-B and UV-C regions; therefore, the photo-protective property can be expected. Photostability of gadusol was evaluated by irradiating a steady monochromatic light and no additional bands appeared [23]. The photodecomposition quantum yields are very small, even in the presence of oxygen (approximately 4 × 10^−2^ for gadusol and 1 × 10^−4^ for gadusolate). Gadusol and gadusolate show no fluorescence, and thus the photoacoustic calorimetry was studied. The results clearly show that rapid non-radiative decay is the dominant relaxation pathway of the excited species at pH 7, which implies a UV-sunscreening role of gadusolate. Additionally, laser flash photolysis experiments proved the electron transfer reaction of the ground state of gadusolate with some triplet sensitizers: benzophenone, acridine, rose bengal, in water or methanol solution. A rate constant for the quenching of rose bengal triplet state is (2.0 ± 0.1) × 10^8^ M^−1^·s^−1^ in water at pH 7, which might suggest inhibition of generating singlet oxygen.

### 2.2. 4-Deoxygadusol (Figure 2(1b))

The structure of 4-deoxygadusol (DG) was firstly determined as an unstable hydrolysate from mycosporine-glycine (M-Gly) isolated from the zoanthid *Palythoa tuberculosa* [24]. DG was obtained easily by heating an M-Gly solution at 80 °C for 3 h. On the other hand, the methyl ester of M-Gly was relatively stable, indicating a contribution from the intramolecular carboxylate anion on the hydrolysis process. Both ^1^H and ^13^C NMR spectra were completely consistent with a symmetrical structure. An absorption maximum in acidic condition reached at 268 nm (enol form) and in basic condition the maximum appeared at 294 nm (enolate form). Thereafter, DG was also isolated from various fish eggs of *Scomber scombrus*, *Auxis thazard*, *Belone belone*, *Box boops*, and *Trachurus trachurus* [17] and *Urchin* eggs [25]. DG has been recognized as a precursor for MAAs and not as an artifact; thus, significant research on DG biosynthesis has been carried out.

Studies of the antioxidant properties of DG have been more rare. An inhibition assay for phosphatidylcholine peroxidation (PC-OOH) was first pursued by Dunlap *et al.* The assay revealed stronger inhibition properties of DG relative to shinorine and M-Gly [26]. A recent study of singlet oxygen quenching by DG revealed significantly stronger quenching compared with a well-known ^1^O_2_ quencher [27]. The rate constant for quenching ^1^O_2_ by DG (k_Q_) was determined by the steady-state method based on competitive inhibition of rubrene oxidation and was found to be 5.4 × 10^7^ M^−1^·s^−1^, which is comparable to the k_Q_ value of M-Gly and is higher than those of furfuryl alcohol and 1,4-diazabicyclo[2,2,2]octane (DABCO).

## 3. Mono-Substituted MAAs (Aminocyclohexenone-Type MAAs, Figure 3)

**Figure 3:**
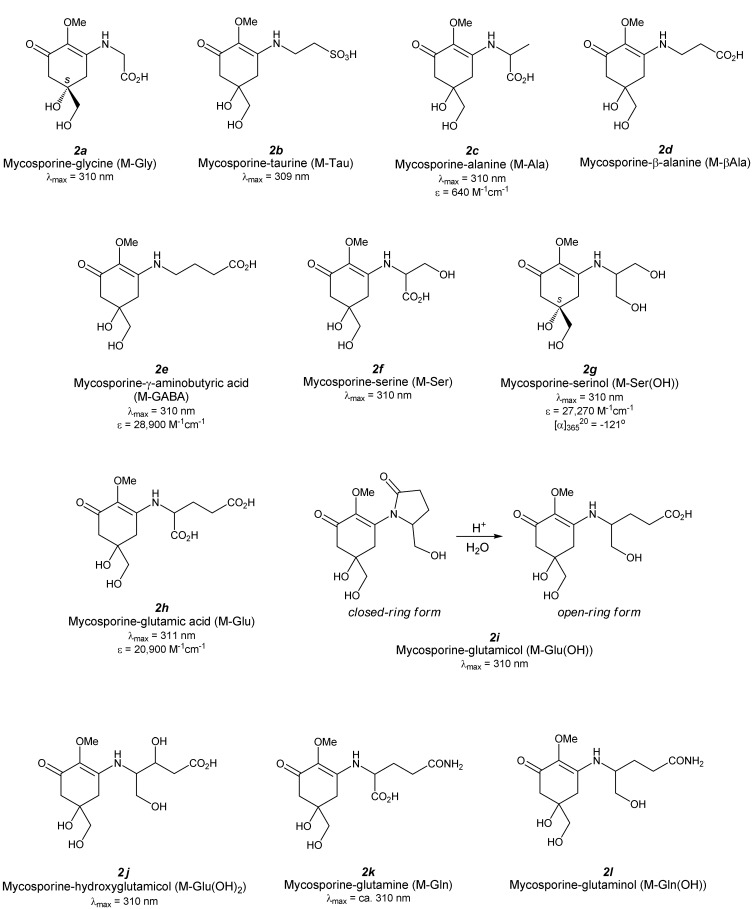
Chemical structure and spectroscopic properties of aminocyclohexenone-type MAAs. Abbreviations are shown in parenthesis.

### 3.1. Mycosporine-Glycine (Figure 3(2a))

Mycosporine-glycine (M-Gly) was first found in the zoanthid *Palythoa tuberculosa* at the same time that deoxygadusol was identified [24] and isolated by repeated column chromatography of aqueous ethanol extract to obtain a pale yellow amorphous powder. After, M-Gly was found in various microalgae and cyanobacteria [28]. The structure, determined from NMR and mass spectra, revealed a glycine-substituted cyclohexenone substructure. M-Gly can be synthesized from D-(−)quinic acid through fifteen steps in a very low overall yield of approximately 1% [29]. Optical rotation ([α]_D_) of its methyl ester is −11.3°, which is close to the methyl ester of the naturally occurring M-Gly −12°). This result revealed that the stereogenic center of M-Gly can be defined as an *S*-form and agrees with the proposed biosynthesis of M-Gly via the shikimate pathway. The absorption maximum of M-Gly is 310 nm, which red-shifted from its precursor, deoxygadusol.

The ability of M-Gly to inhibit lipid peroxidation was first investigated by using the phosphatidylcholine (PC) peroxidation assay [30]. M-Gly inhibits PC-OOH formation in a concentration-dependent manner and shows moderate activity at 30 mM. However, the molar antioxidant efficiency of M-Gly is remarkably less than those of l-ascorbic acid and uric acid, which are water-soluble antioxidants. The moderate inhibition activity is also confirmed by a β-carotene bleaching method [31]. The percentage of inhibition of β-carotene bleaching compared to α-tocopherol as a hydrophobic antioxidant becomes large as the M-Gly concentration increases. The absolute value of the activity, however, is only 5% that of α-tocopherol at the same concentration.

The radical scavenging capacity of M-Gly was investigated by using a hydrophobic, artificial, stable radical: DPPH radical [32]. The radical scavenging activity of M-Gly increased with its concentration. M-Gly showed a comparable half maximal inhibitory concentration (IC_50_) value (4.23 ± 0.21 mM) to that of ascorbic acid (3.12 ± 0.18 mM). The reaction with water-soluble ABTS radical has been thoroughly investigated [31]. The percentage of scavenging became larger as the M-Gly concentration increased, and this behavior was independent of the pH value, though the absolute scavenging percentage increased as the pH value increased. The IC_50_ value was summarized in Table 1 and the highest activity was obtained in basic medium (pH 8.5). This behavior is different from that of L-ascorbic acid, a common water-soluble antioxidant, which shows maximum scavenging in neutral medium (pH 7.5). However, the scavenging power of M-Gly and ascorbic acid are comparable. Scavenging of superoxide generated by the pyrogallol system was studied; however, no scavenging reaction was observed for M-Gly.

**Table 1:** pH dependence of IC_50_ values on ABTS radical scavenging by various MAAs.

pH Value	M-Gly	A-330 (+Pi 14%)	P-334 (+SH)	SH	Asc
6.0	20	1000	1000	–	11
7.5	4	60	400	–	4
8.5	3	10	80	100	26

M-Gly: mycosporine-glycine Figure 3(2a), A-330: asterina-330 Figure 4(3g), P-334: porphyra-334 Figure 4(3a), SH: shinorine Figure 4(3e), Pi: palythine Figure 4(3d), Asc; l-ascorbic acid, –: not determined (too large).

The capacity of ^1^O_2_ quenching of M-Gly has been studied in the presence of eosin Y (EY) as a photosensitizer [33]. A competitive reaction of M-Gly with ^1^O_2_ against 2,2,6,6-tetramethyl-4-piperidone (TMPD) was performed in a concentration-dependent manner. Samples of either M-Gly alone or M-Gly plus TMPD did not give a nitroxide radical. Degradation of M-Gly by ^1^O_2_; formed by the EY system occurred in aerobic conditions, though no degradation was seen in anaerobic (N_2_ purged) conditions and degradation of M-Gly was partially cancelled by the addition of sodium azide as a ^1^O_2_ quencher. This result indicates quenching of ^1^O_2_ by M-Gly is mainly achieved via a chemical mechanism; however, the result does not eliminate the contribution of physical mechanism. Three major reaction products were detected by HPLC; however, the structures are still unknown. In contrast, photolysis of M-Gly alone did not give any degradation products. This result indicates M-Gly does not undergo self-photosensitized degradation; nevertheless UV absorbance of M-Gly is very large. Analysis of the time-resolved phosphorescence decay results by the Stern-Volmer equation revealed the fast quenching reaction constant by M-Gly (k_Q_ = 5.6 × 10^7^ M^−1^·sec^−1^). It is comparable to the values for some well-known ^1^O_2_ quenchers under the same experimental conditions, such as DABCO and α-tocopherol.

The protective effect of M-Gly, isolated from *Scallop ovaries*, on human lung fibroblast (WI-38) cells from UV-induced cell death was investigated [34]. M-Gly strongly protects cells from UV-induced cell death in a concentration-dependent manner and the half maximal effective concentration (EC_50_) value for M-Gly is 24 µM, which is larger than those of the aminocyclohexene-imine type MAAs SH and P-334. The viability rate was reduced to the control levels when the medium containing MAAs was changed to medium without MAAs before UV irradiation. Growth promotion activity of M-Gly on normal human skin fibroblast, TIG-114 cells. M-Gly did not suppress cell growth at all and proliferation rate is increased in a concentration-dependent manner. 50 µM M-Gly accelerated growth by approximately 40% of the cell proliferation. Thus, the protective effect of M-Gly against UV-induced cell death may be due to UV-screening and cell proliferation properties.

### 3.2. Mycosporine-Taurine (Figure 3(2b))

Mycosporine-taurine (M-Tau) was isolated using an aqueous methanol (80%) extract of sea anemone *Anthopleura elegantissima*, and its structure was identified by acid hydrolysis, followed by amino acid analysis and LC/MS/MS measurement [35]. However, its precise structure was not determined by NMR analyses. Its absorption maximum is 309 nm. UV-A, -B, and -C irradiation to M-Tau for one hour resulted in photodecomposition regardless of the wavelength difference. Treatment of hydrogen peroxide also degraded M-Tau very efficiently; indicating that M-Tau can act as a potential antioxidant [36].

### 3.3. Mycosporine-Alanine (Figure 3(2c))

Mycosporine-alanine (M-Ala) was identified as a self-germination inhibitor from the conidial mucilage *Colletotrichum graminicola* [37]. Its structure determination was performed via high-resolution mass spectrometry of trimethylsilylated M-Ala. The exact mass number revealed that mono-trimethylsilylated compound (found: 332.1526, calculated: 332.1529) and thus molecular formula of M-Ala was C_11_H_17_O_6_N, which has a molecular weight of 259.1051. This molecular formula is consistent with the structure for M-Ala. Other spectral data for structure determination has not been reported. The absorption maximum of M-Ala is 310 nm and the extinction coefficient is 640 M^−1^·cm^−1^, which is strangely much smaller than those of other aminocyclohexenone-type MAAs.

### 3.4. Mycosporine-β-Alanine (Figure 3(2d))

Mycosporine-β-alanine (M-βAla) was isolated from the 70% ethanol extract of sea anemone *Radianthus* sp. and was reported in an industrial patent [38]. Its structure was determined by MS and 1D-NMR measurements, though follow-up treatises have never been reported.

### 3.5. Mycosporine-γ-Aminobutyric Acid (Figure 3(2e))

Gamma amino acid substituted MAA (M-GABA) was recently reported for the first time [39]. Aqueous methanol (30%) extract from *Nostoc commune* was subjected to several types of chromatography and the purified compound was analyzed by spectroscopic methods. High-resolution mass spectroscopy (HRMS), matrix-assisted laser desorption/ionization time-of-flight mass spectroscopy (MALDI-TOF/MS/MS), 1D- and 2D-NMR, IR measurements revealed that the aminocyclohexenone-type MAA substructure was comprised of γ-aminobutyric acid via an imine-linkage. The absorption maximum is 310 nm with an absorption coefficient of 28,900 M^−1^·cm^−1^.

The scavenging capacity of M-GABA against water-soluble ABTS radical was tested and its strength was comparable to those of ascorbic acid and Trolox, which are typical antioxidants. This result means that the radical scavenging capacity of M-GABA is similar to that of M-Gly because both MAAs have aminocyclohexenone-type substructures.

### 3.6. Mycosporine-Serine (Figure 3(2f))

Mycosporine-serine was reported in 1979 from extract of *Stereum hirsutum* [40]. Its absorption maximum at 310 nm indicates that it is an aminocyclohexenone-type MAA with an amino acid. NMR and mass spectral analysis revealed a serine derivative of MAA. Mycosporine-serine (M-Ser) might not so common because only a limited example of the isolation has been reported. The other example was reported by Lunel *et al.*, who confirmed M-Ser from *Pyronema omphalodes* [41].

### 3.7. Mycosporine-Serinol (Figure 3(2g))

Mycosporine-serinol (M-Ser(OH)) was identified in fungi as a UV-absorbing substance associated with light-induced sporulation [42]. Its structure was determined by the combination of several spectroscopic analyses; UV-VIS, IR, MS, and NMR spectra [43] and these analyses indicated an aminocyclohexenone-type MAA substructure and reduced serine; serinol, via an imine-linkage. The absolute configuration of M-Ser(OH) was revealed as an *S*-form according to the same process for determining the configuration of M-Gly [29,44]. The absorption maximum is 310 nm with an extinction coefficient of 27,270 M^−1^·cm^−1^. Specific rotation of M-Ser(OH) at 365 nm ([α]_365_^20^) is −121°.

Photolysis of M-Ser(OH) in the presence of photosensitizer flavin had been investigated and slow photodegradation occurred [45]. Reaction of M-Ser(OH) with singlet oxygen can be expected, though sufficient experimental detail has not yet been exhibited.

### 3.8. Mycosporine-Glutamic Acid (Figure 3(2h))

Mycosporine-glutamic acid (M-Glu) had been isolated from *Glomerella cingulata* [46]. An amino acid substituent was determined by hydrolysis of purified M-Glu in boiling water for five hours and the MAA structure was confirmed by MS and 1D-NMR spectra. M-Glu was slightly unstable on cellulose and silica gel to give hydrolysate. Its absorption maximum is 311 nm in water with an extinction coefficient of 1 × 10^4.32^ (*ca.* 20,900) M^−1^·cm^−1^.

Photolysis of M-Glu in the presence of photosensitizer flavin had been investigated and fast photodegradation occurred in air-saturated condition under white light [45]. Reaction of M-Glu with singlet oxygen can be expected as same as glutamine derivative (M-Gln), though experimental detail was not exhibited unlike M-Gln.

### 3.9. Mycosporine-Glutamicol (Figure 3(2i))

Mycosporine-glutamicol (M-Glu(OH)), originally called mycosporine-2, had been isolated from *Pyronema omphalodes* [41] and *Gnomonia leptostyla* [47] as an open ring form. It can be easily synthesized by the acid hydrolysis of closed-ring form, cyclic amide, though it is not thought as an artifact formed during purification. HPLC results showed the presence of an open-ring form, *ca.* 5% in *G. leptostyla*. It can also be found in *Botrytis cinerea* in which a little of the open-ring form was also found, though the amount in the aged cultures was larger than in the young cultures [48]. The structure was determined from the results of NMR and MS spectra, but its absolute configuration has not been clarified yet. M-Glu(OH) has an absorption maximum at 310 nm.

Slow photodegradation of M-Glu(OH) occurred in the presence of photosensitizer flavin, which suggests the reaction of M-Glu(OH) with singlet oxygen, but insufficient experimental detail was exhibited [45].

### 3.10. Mycosporine-Hydroxyglutamicol (Figure 3(2j))

Mycosporine-hydroxyglutamicol (M-Glu(OH)_2_) has been recently found in the extract from two lichens, *Ddegelia plumbea* and *Nephroma laevigatum* [49]. The researchers applied HPLC coupled to spectrophotodensitometry, HPLC-DAD-MS^n^ and UPLC-HRMS analyses to the screening of new MAAs in lichen and found eight unknown MAAs. The structure of the purified sample of one of these MAAs was analyzed by HRMS and 1D- and 2D-NMR and they revealed the structure as an aminocyclohexenone-type MAA with hydroxylglutamicol as a side chain. The absorption maximum was found to be 310 nm. The absolute configuration, cyclization status, and other physical and chemical properties are unknown at present.

### 3.11. Mycosporine-Glutamine (Figure 3(2k))

Mycosporine-glutamine (M-Gln) was isolated from the EtOH extract of the fungi *Pyronema omphalodes* and *Glomerella cingulata* [50]. Its structure was determined by MS spectrometry. Hydrolysis of M-Gln on a cationic resin at 40 °C for 20 h gives M-Glu, which was assigned by comparing the HPLC results with purified M-Glu. M-Gln has an absorbance at approximately 310 nm.

Photolysis of M-Gln was investigated. M-Gln did not decompose in an N_2_ atmosphere, although in air-saturated condition photodecomposition was enhanced [45]. The degradation reaction rate of M-Gln in the presence of flavin as a photosensitizer further increased. This result indicates that M-Gln reacts with singlet oxygen. Flavin-sensitized photodecomposition in the N_2_ saturated condition should be tested as a negative control experiment; however, singlet oxygen quenching by M-Gln might be certain because the disappearance of M-Gln was delayed by the addition of DABCO as a singlet oxygen quencher.

### 3.12. Mycosporine-Glutaminol (Figure 3(2l))

Mycosporine-glutaminol (M-Gln(OH)) was isolated from several *Deuteromycetes* species including *Trichothecium roseum*. The structure of M-Gln(OH) was determined by MS and NMR spectrometry [51,52]. M-Gln can be hydrolyzed at pH 2 and 100 °C to give 4-DG, glutaminol, M-Glc(OH) and glutamicol, which agree well with the determined structure of M-Gln(OH).

Slow photodegradation of M-Gln(OH) occurred in the presence of a photosensitizer flavin, which suggests the reaction of M-Gln(OH) with singlet oxygen; however, additional experimental detail is required [45].

## 4. Di-Substituted MAAs (Aminocyclohexene Imine-Type MAAs, Figure 4)

**Figure 4:**
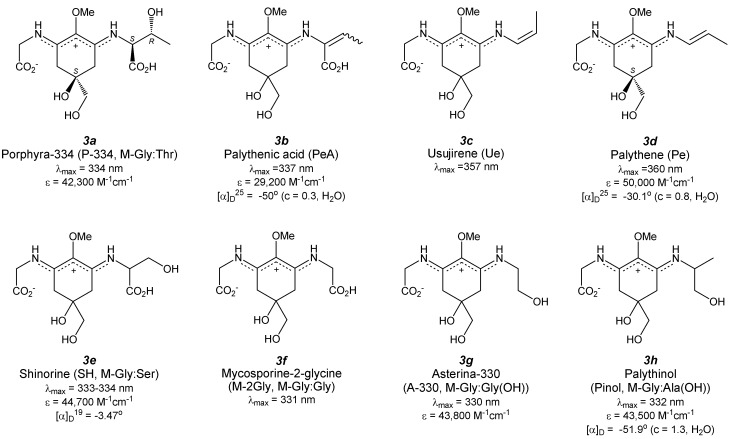

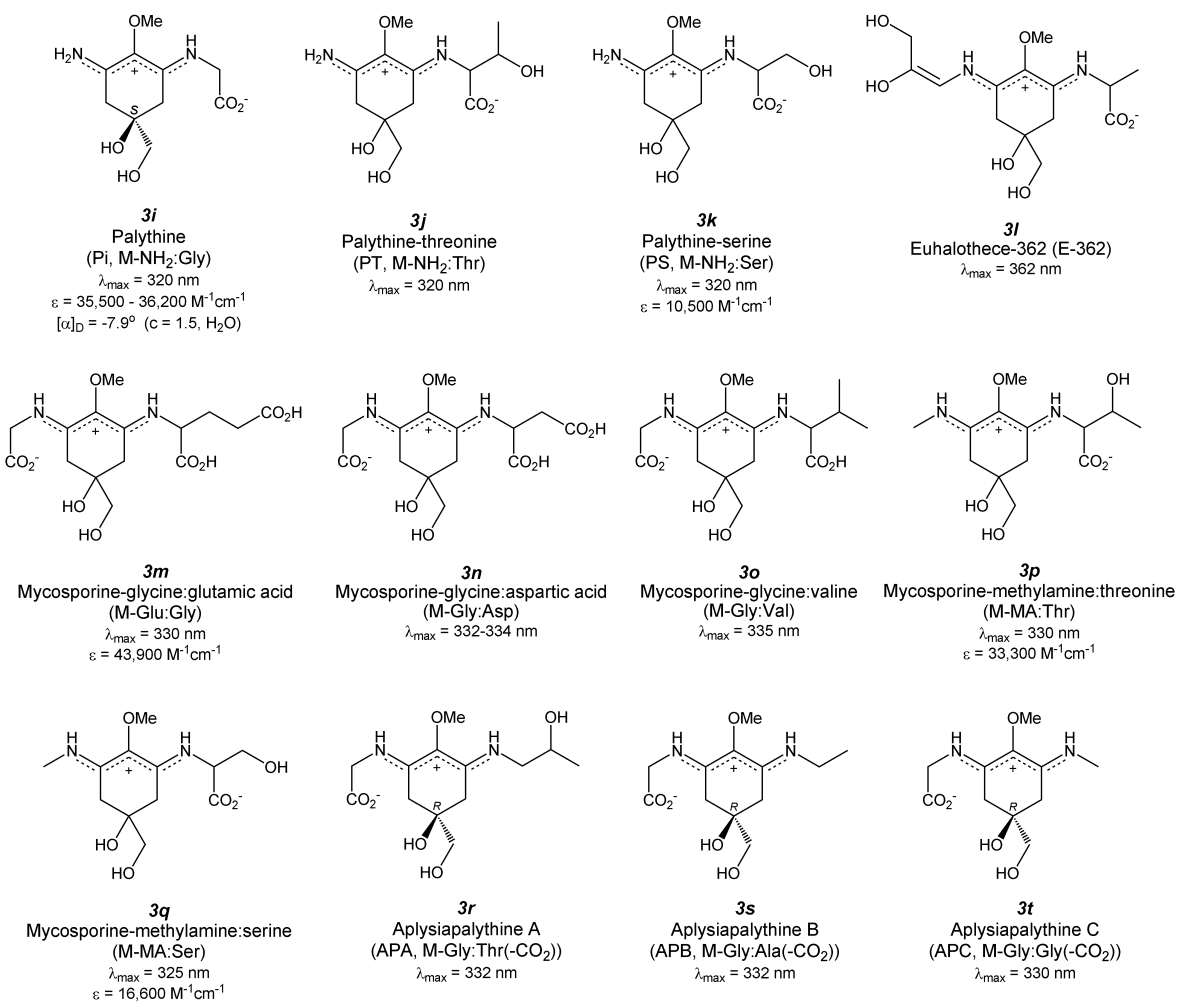
Chemical structure and spectroscopic properties of aminocyclohexene imine-type MAAs. Abbreviation is in parenthesis.

### 4.1. Porphyra-334 (Figure 4(3a))

Porphyra-334 (P-334) was first identified in a red alga *Porphyra tenera* Kjellman [53]. Dried alga was extracted with 80% methanol and the extract was purified by several applications to column chromatography to give colorless powder. At almost same time, isolation was reported from *Palythoa tuberculosa* [54] and *Mytilus galloprovincialis* [55]. The structure of P-334 can be determined by NMR, IR, MS and absorption spectra and identified as an aminocyclohexene imine backbone possessing two amino acids: threonine and glycine, at both ends. The absolute configuration of P-334 has been recently clarified by applying various NMR techniques and computer simulation methods. The absolute configuration of threonine residue was resolved by nuclear overhauser effect correlated spectroscopy (NOESY) and was supported by *ab initio* calculations [56]. The configuration of threonine is that of the *L*-form, similar to the natural amino acid. The stereogenic center of the central aminocyclohexene imine is the *S*-form, which agrees well with the absolute configuration of M-Gly [29] and the supposed configuration of the proposed biosynthetic pathway. P-334 has a large absorption band at 334 nm (ε = 42,300 M^−1^·cm^−1^), which is red-shifted relative to the carbonyl-type MAAs. This absorption band can be slightly blue-shifted and its intensity can be slightly decreased in a highly acidic medium (pH < 3) at room temperature; however, this change is reversible when the pH is adjusted to a higher value [57,58]. In contrast, the absorption band immediately and irreversibly decreases in a highly basic medium (pH > 12.0) at room temperature. Thermal stability of P-334 isolated from the extract of *Gracilaria cornea* was studied [59] and the absorption spectrum was not changed by the long-term heat treatment (75 ± 2 °C, 6 h). The photophysical properties of P-334 have been thoroughly investigated by Conde *et al.* [60]. The photodecomposition quantum yield of P-334 in an aqueous air-saturated solution was shown to be very low (2.4 ± 0.3 × 10^−4^), which indicates the great photostability of P-334. However, P-334 can be photo-decomposed at a rate constant of 4.98 × 10^−3^ m^2^·kJ^−1^only when sensitized by a strong sensitizer such as riboflavin in the presence of oxygen [61]. Conde *et al.* measured the fluorescence spectrum of P-334 and a very weak emission band was observed with a maximum at 395 nm after excitation at 334 nm [60]. A relative fluorescence quantum yield was determined from the spectrum of quinine bisulfate and was estimated at 2.0 ± 0.2 × 10^−4^. The fluorescence decay was obtained by the time-correlated single photon counting method. The decay kinetics can be fitted to an exponential function and a lifetime of the excited-singlet state of P-334 was determined to be 0.40 ± 0.05 ns. Laser flash photolysis experiments at 355 nm did not show any transient species. The triplet state of P-334 was sensitized by triplet-triplet (T-T) energy transfer from various photosensitizers, including benzophenone, biphenyl, 1-naphtalene-methanol, and phenanthrene. The absorption band to the triplet state of P-334 has an absorption maximum at 440 nm (ε = 1.0 ± 0.2 × 10^4^ M^−1^·cm^−1^). The lifetime of the triplet state is 14 µs and the maximal quantum yield of absorption to the triplet state is 0.030 ± 0.005. These photophysical properties of P-334 can be explained by a very fast internal conversion process, which supports the photoprotective role of P-334. Photoacoustic calorimetry experiments revealed the non-radiative relaxation pathways of the excited P-334 in an aqueous solution; almost all of the absorbed energy (*ca.* 97%) is promptly delivered to the surroundings as heat [62]. Due to this photostability, P-334 is a potential candidate for biochemical-electrical devices such as the field effect transistor (FET). The photoelectrical properties of P-334 under UV-irradiation have been studied very recently [63]. Low photocurrents can be observed when the gate voltage is under −3.0 V (3.20 × 10^−6^ A in the dark, 3.92 and 3.95 × 10^−6^ A under illumination at 254 and 365 nm, respectively); however, the photocurrent can be drastically increased under illumination at 334 nm (8.83 × 10^−6^ A). Repeated measurements reveal similar photo-responses with the same values of photocurrent, which suggests no photo-remittance in the device upon repeated illumination and potential applications for FET devices made from MAAs.

The aqueous extract from *Porphyra tenera* containing mostly P-334 shows moderate inhibitory effects on the rate of phosphatidylcholine peroxidation induced by 2,2′-azobis(2-amidinopropane) dihydrochloride (AAPH) [30]. The inhibition capacity was similar to the extract from *Lissoclinum patella* containing M-Gly and shinorine (30% and 70%, respectively), though it was lower than the extract from *Palythoa tuberculosa* containing M-Gly (66%). This result indicates that the aminocyclohexenone-type MAA might show stronger lipid peroxidation inhibition properties than aminocyclohexene imine-type MAAs. HPLC analysis during the lipid peroxidation reaction reveals P-334 is almost unreactive against oxidation. In contrast, a recent study by Tao *et al.* reported moderate inhibition properties of purified P-334 against peroxidation of linoleic acid [64]. Furthermore, same authors investigated the synergetic effects of α-tocopherol and P-334. The simultaneous application of 0.02 µM α-tocopherol and 50 µM P-334 to the reaction system effectively suppressed the oxidation level to approximately 40% of a single application of P-334. Singlet oxygen-induced hydroperoxide formation was also suppressed by P-334; this indicates the possibility of quenching singlet oxygen similar to M-Gly. Lipid peroxide inhibition determined by the β-carotene bleaching method also demonstrates the moderate activity of the MAA mixture containing P-334 and a small amount of SH [31].

Two research groups studied the radical scavenging activities of P-334 against DPPH radicals and found very low reactivity of P-334 [32,65], unlike the carbonyl-type MAAs such as M-Gly. The reactivity of low-molecular weight extract from *Porphyra yezoensis* increases as temperature does; this indicates heat treatment, including cooking of food containing MAA has a way to improve radical scavenging properties [65,66]. The extract contains P-334; thus, heat treatment of purified P-334 at 120 °C for 30 min has been performed and identification of the decomposed product by MS and NMR analyses revealed its dehydrated derivative, whose IC_50_ value (10.1 ± 0.2 µg/mL) was much less than that of P-334 (>1000 µg/mL). These results mean that dehydration of P-334 increases radical scavenging activity. The chemical structure of the dehydrated P-334 was proposed; however, further measurements are necessary for complete structure determination. Detectable DPPH radical scavenging by arabinose substituted P-334 (P-334-Ara) was not observed [39,67], which is consistent with results from previous reports [32,65]. De la Coba *et al.* investigated the reaction of P-334 containing shinorine as an impurity with water-soluble ABTS radical and found lower reactivity than M-Gly [31], which is consistent with the result of DPPH radical scavenging. The dependence of IC_50_ on pH value was summarized in Table 1. The IC_50_ value became lower in a basic medium (pH 8.5) than in an acidic medium (pH 6.0). The amplification efficiency (the ratio of IC_50_ values) of P-334 is 12.5, which is twice as much as that for M-Gly (6.7). This result indicates that the aminocyclohexene imine-type MAA might play a very important scavenging role especially under basic conditions, though aminocyclohexenone-type MAA functions over a wide pH range. This strong pH dependence of ABTS radical scavenging by P-334 will be derived from its zwitterionic form. P-334 preferentially exists in a negatively charged form at pH 8.5 and an almost neutral form at pH 6 because the p*K_a_* value of P-334 is 5.7 [68]. The conjugated amino imine form is therefore more susceptible to hydrogen atom donation than the cation charged delocalized form (dominant in an acidic medium). The water-soluble ABTS radical scavenging capacities of glycosylated P-334 derivatives were also tested. Comparing activities of P-334-Ara and P-334-Man with that of P-334 and the typical antioxidants such as ascorbic acid and Trolox is difficult because the dependence of the scavenging capacity on pH has not been investigated, though P-334-Ara and P-334-Man certainly quench the ABTS radical [39,69].

P-334 shows significant antioxidant activity against superoxide radicals at a concentration greater than or equal to 200 µM [31,70]. Approximately 46% inhibition was observed when the concentration of P-334 was 1 mM.

The low quantum yield of excitation to triplet state can exclude the possible role of P-334 as a photosensitizer to generate ^1^O_2_. Although the photodecomposition quantum yield increases in the presence of oxygen, it remains on the order of 10^−4^. Therefore, P-334 does not generate reactive intermediates such as radical species and/or other oxidizing species including ^1^O_2_ during irradiation [60]. The effect of P-334 on singlet oxygen-induced lipid-hydroperoxide formation was evaluated by the FOX1 method [64]. P-334 prevents lipid peroxidation effectively in a concentration-dependent manner. This result indicates that singlet oxygen is quenched by P-334, though there is a potential risk of over-estimation because P-334 inhibits lipid peroxidation. In contrast, Whitehead *et al.* studied the degradation profile of P-334 in the presence of riboflavin as a sensitizer in the presence of oxygen. P-334 can be certainly photo-decomposed, though the researchers proposed the major photosensitized degradation pathway of P-334 is type-I photo-oxidation [61]. However, this does not exclude possibility of singlet oxygen quenching capacity of P-334. To overcome this problem, it will be necessary to evaluate the quenching of singlet oxygen without photosensitization, for example via thermal decomposition of endoperoxide.

UV-absorbing compounds from *Porphyra yezoensis* contain the three major MAAs palythine, shinorine, and porphyra-334, which very efficiently prevent the formation of thymine dimers, an important structure of UV-induced DNA damage, under illumination with a Xe lamp [71]. The mechanism of this photo-protection can be assumed to involve energy transfer from the excited thymine residues to MAA (mechanism unknown) and/or a filtering effect from UV radiation.

Recent studies have revealed the protective effect of P-334 against the acute negative effects of UV radiation on controlling the anti-oxidative defense system, cell viability and apoptosis. Schmid *et al.* reported the UV-A protective effect of the extract from *Porphyra umbilicalis* containing P-334 and a small amount of SH [72]. After UV-A irradiation, the growth of mouse fibroblast 3T3 cells was reduced compared to non-treated cells. The presence of MAAs in the culture medium protects the cells in a concentration-dependent manner from growth-inhibition induced by UV-A irradiation. Application of MAAs on the skin of women between the ages of 36 and 54 to combat UV-A exposure resulted in improvements in skin firmness, smoothness and wrinkle depth. De la Coba *et al.* investigated the effect of application of an MAA mixture consisting of P-334 and SH extracted from *Porphyra rosengurttii* on murine skin [73]. Animals treated topically with P-334 + SH showed a lower redness level of skin and edema formation in response to UV light than untreated animals. Levels of heat shock protein (Hsp70) were estimated in epidermal tissues of MAA-treated and untreated mice and Hsp70 was significantly overexpressed at just 6 h after UV exposure in untreated tissue, though expression in treated mice was greatly suppressed. Later, untreated tissue showed progressive decreases, while treated tissue showed temporal increase and recovery to a basal level. The activity of superoxide dismutase (SOD) after UV illumination gradually decreased both in MAA-treated and untreated tissues, though MAA-treated tissue maintained higher levels than untreated tissue for 72 h. Catalase activity in MAA-treated tissue did not change for 72 h; however, untreated tissue showed a gradual decrease in activity. The effect of the extract from *Porphyra yezoensis*, consisting of P-334 and SH as major MAA species on the UVB-exposed HaCaT cells (human keratinocytes) has been very recently investigated [74]. UVB exposure to HaCaT cells results in a decrease in viability and an increase in apoptotic cells, concomitantly decreasing the total glutathione content and the ratio of reduced and oxidized glutathione (GSH/GSSG). Post-treatment with the extract to the damaged cells significantly increases the net viability, apoptotic cell fractions, and GSH/GSSG ratio. The treatment enhances the expression of *p*-JNK and *p*-ERK. These results suggest that the post-treatment with P-334 (+SH) protects UVB-exposed skin cells not only by increasing cell proliferation but also by inducing apoptosis of damaged cells, activating JNK and ERK signaling pathways. The modulation of the total content and redox balance of glutathione may contribute to these pathways.

The effect of purified P-334 has been recently studied. P-334 isolated from *Porphyra yezoensis* suppressed ROS production and the expression of matrix metalloproteinase following UVA irradiation of human skin fibroblasts, while increasing levels of extracellular matrix components such as procollagen, type I collagen, elastin [75]. Suh *et al.* investigated the effect of P-334, isolated from *Chlamydomonas hedleyi*, on inflammation and skin aging of HaCaT cells [32]. P-334 had no effect on cyclooxygenase-2 (COX2) expression up to a concentration of 300 µM, which plays a key role in the generation of inflammatory responses and the expression of which is increased in inflamed tissue. However, the expression (mRNA) levels of some genes, which correlate with skin aging caused by UV irradiation, had been changed. In the presence of P-334, suppression levels of procollagen C-endopeptidase enhancer (PCOLCE) and elastin rebounded in a concentration-dependent manner to those in UV-untreated cells. Interestingly, elastin was more highly expressed in cells treated with P-334 than in UV-untreated cells (via an unknown mechanism). In contrast, the involucrin level remains high even when P-334 was applied. These results indicate potential applications of P-334 in cosmetics because of its protecting activities from skin aging caused by UV irradiation.

### 4.2. Palythenic Acid (Figure 4(3b))

Dehydration of the threonine chain in P-334 gives palythenic acid (PeA). PeA was isolated from the ascidian *Halocynthia roretzi* in a very low yield of 0.0006% via several types of column chromatography [76]. The structure of the purified amorphous colorless powder having a melting point (mp) at 153–156 °C was identified by 1D-NMR spectroscopy. The configuration around the double bond in dehydrated threonine groups (*E* and *Z* isomers) can be recognized from their chemical shifts; the chemical shift values of methyl and olefinic protons of the *Z* isomer appear at higher and lower field regions than those of the *E* isomer, respectively. UV absorption spectrum of PeA exhibits the absorption maximum at 337 nm, which is slightly red-shifted relative to that of P-334, and it displays a moderate molar extinction coefficient (ε = 29,200 M^−1^·cm^−1^). Recent applications of reverse-phase HPLC suggest the possibility of separating these isomers [77]; however, the procedure still presents difficulties for complete separation. A photo-degradation rate constant of PeA sensitized by riboflavin in aerobic conditions was found to be 5.52 × 10^−3^ m^2^·kJ^−1^, which is higher than that of P-334 [61], potentially because of the nucleophilic character of the double bond.

### 4.3. Usujirene (Figure 4(3c)) and Palythene (Figure 4(3d))

Decarbonylation from *Z*- and *E*-palythenic acid gives usujirene (Ue) and palythene (Pe) possessing *cis*- and *trans*-double bonds. Pe was purified by repeated chromatography and preparative TLC from the aqueous ethanol extract of *Palythoa tuberculosa* to give yellow crystals (mp = 145–146 °C, [α]_D_ −30.1° (*c* = 0.8 in H_2_O)) [78]. Its structure was determined by ^1^H-NMR, ^13^C-NMR, and IR spectra, along with elemental analysis. X-ray crystallographic analysis revealed absolute configuration of Pe to be the *S*-isomer [79]. The absorption maximum appeared at 360 nm, which is one of the most red-shifted bands in the known MAA species. This red-shift may result from the conjugated structure of π-electrons. The molar absorption coefficient of Pe is 50,000 M^−1^·cm^−1^. The other cis-form isomer was isolated from the red alga *Palmaria palmata* and was named usujirene (Ue) [80]. 1D- and 2D-NMR, IR, and mass spectra were used to determine the structure of Ue and the *cis*-form of the double bond geometry. The absorption maximum of Ue is 357 nm (unknown molar absorption coefficient), which is slightly blue-shifted relative to Pe. A recent study revealed the phototransformation of Ue to Pe in aqueous solution [81]. The quantum yield for the photo-isomerization process from Ue to Pe is determined to be (1.71 ± 0.13) × 10^−5^, though the yield for the reverse process is ten times less efficient ((1.37 ± 0.15) × 10^−6^). The isomerization process achieves a photostationary state when almost 8% of the isomers in the mixture are Ue. This might explain why several dinoflagellate species accumulate Pe preferentially *in vivo*. Photosensitized degradation of Pe sensitized by riboflavin in aerobic conditions has been investigated [61]. The rate constant is estimated as 36.8 × 10^−3^ m^2^·kJ^−1^, which is one order of magnitude larger than those of several aminocyclohexene imine-type MAAs such as palythine, palythinol, shinorine, porphyra-334, palythenic acid, and asterina-330. This increased rate constant may be attributed to the extra double bond in the side chain, which would be attractive to electrophiles such as hydroxyl or peroxy radicals.

When Pe was boiled with 2N HCl for 3 h, it afforded palythine (Pi) and propanal. Acid-catalyzed hydrolysis occurs at the propene side group, not at the glycine side. Proton transfer might occur from the iminium cation to the electrophilic double bond. Hydrogenation of Pe with Pd-C as a catalyst in H_2_O for 1 h affords dihydro-palythene, whose absorption maximum appears at 331 nm. This result implies that the propene side group can be reduced first and the MAA backbone is not reduced in this condition.

Nakayama *et al.* isolated Ue from *Porphyra yezoensis* and investigated the inhibition property against lipid peroxidation [82]. Lipid peroxide made from linoleic acid has been quantified by using the ferric-thiocyanide method and malondialdehyde has been quantified by using the thiobarbituric acid method. Both methods reveal stronger activity of Ue than α-tocopherol. This high antioxidant activity might be explained by the donation of a hydrogen atom from C-4, C-6, or C-9 methylene to the lipid peroxide radical. It is because the free radical that formed in Ue can be stabilized by resonance with double bonds, as seen in α-tocopherol.

### 4.4. Shinorine (Figure 4(3e)) and Its Methyl Ester

Shinorine (SH) was first found in the edible mussel *Mytilus galloprovincialis* in an inseparable mixture with P-334 [55]; then, it was isolated from the extracts of *Chondrus yendoi* [83] and *Trichocarpus crinitus* [84]. Purified SH shows a melting point at 154–156 °C, and the specific rotation ([α]_D_^19^) is –3.47°. The structure was determined using spectral techniques such as NMR, IR, MS, and absorption spectra, and the structure was identified as an aminocyclohexene imine backbone possessing the two amino acids serine and glycine at both ends. As far as the authors know, the absolute stereostructure of SH has not been clarified yet. The addition of methanol to syrupy SH concentrate gives white crystals [85], although X-ray crystallographic analysis has never been performed. The absorption spectrum of SH shows an absorption maximum at 333–334 nm with an extinction coefficient of 10^4.65^ (*ca.* 44,700) M^−1^·cm^−1^, which are comparable to those of P-334. The thermal stability of the SH extract from *Gracilaria cornea* was estimated from the changes in the absorption spectra [59]. The absorption spectrum was not affected by the long-term heat treatment (75 ± 2 °C for 6 h). This means SH has high thermal stability and may contribute to the tolerance against heat stress in a habitat of the host.

Photophysical properties of SH have been recently investigated [62]. The photodecomposition quantum yield of SH in aqueous aerobic solution has been revealed to be very low (3.4 ± 0.5 × 10^−4^), though it is slightly larger than that of P-334. This indicates enough photostability of SH as a sun-screening material. Under photosensitized conditions by the strong sensitizer riboflavin, SH can be photodecomposed with a rate constant of 7.39 × 10^−3^ m^2^·kJ^−1^ in the presence of oxygen [61]. Fluorescence spectrum of SH shows very weak and unstructured emission bands with a maximum at 386 nm from excitation at 334 nm. A relative fluorescence quantum yield was determined by comparing the peak area obtained in the spectrum of P-334 and was estimated to be (1.6 ± 0.2) × 10^−4^. The lifetime of the excited-singlet state of SH in air-saturated conditions was determined by fitting the fluorescence decay kinetics and the result shows a short lifetime (0.35 ± 0.10 ns) that is comparable to that of P-334. Laser flash photolysis experiments at 355 nm did not show any transient species, although the transient absorption with a maximum at 440 nm was observed by sensitization with acetone. The efficiency of triplet-triplet energy transfer from acetone to SH is estimated to be high (*ca.* 50%). This transient absorption spectrum of SH is similar to that of P-334. A lifetime of a triplet state of SH was estimated to be 11 µs and the maximum quantum yield of absorption to the triplet state is 0.05. These photophysical properties of SH are almost same as those of P-334 and can be explained by a very fast internal conversion process. Photoacoustic calorimetry experiments revealed the highly efficient non-radiative relaxation of the excited SH in an aqueous solution. Photoelectrical properties of SH under UV-irradiation have been studied and similar profiles of I-V curves for field effect transistor (FET) has been observed as P-334 [63].

Atypical MAA, originally called M-333, has been found in the extract from some cultures of the toxic red tide dinoflagellates; *Alexandrium catenella* and *Alexandrium minutum* [86], *Alexandrium tamarense* [87], *Heterocapsa triqueta* [88]. Mass spectrum indicated the methyl ester of SH as being the probable candidate, though the fine structure has been very recently revealed by several chemical (hydrolysis and methylation) and physicochemical spectra (mass and NMR spectra) [89]. The researchers isolated two types of isomers and identified as mono-methyl ester of SH at both sides of serine and glycine carboxyl groups. Both have an absorption maximum at 333 nm in methanol. Further information of configuration and physical and chemical properties including antioxidant capacities of these MAA methyl esters have never been reported.

The inhibition property of an 80% aqueous methanol extract from *Lissoclinum patella*, consisting of 70% SH and 30% M-Gly, against phosphatidylcholine peroxidation was studied [30]. The extract shows moderate activity due to the effect of M-Gly, because few decompositions of SH had been observed during the lipid peroxidation reaction. This means SH is oxidatively more stable in the lipid peroxidation reaction than M-Gly. The low reactivity of SH compared to M-Gly and 4-deoxygadusol was confirmed in the following review article [90]. However, the other method proved larger activity of SH against lipid peroxidation than M-Gly [31]. Bleaching of β-carotene due to the reaction with lipid peroxide has been prevented by SH more effectively than M-Gly. The reason for this discrepancy has been still unknown, though the possibility of the reactivation of β-carotene by donating hydrogen atom from aminocyclohexene imine-type MAAs, in other words, antioxidative cross-talk system between carotenoid and MAAs is not negligible. Further investigation including the measurement of redox potential of various MAAs is necessary for the deep understanding. Water-soluble artificial radical (ABTS radical) quenching capacity of SH is very low in acidic and neutral media. Contrary SH can quench ABTS radical dose-dependently in a basic condition, though activity is still much lower than that of M-Gly [31] (Table 1). On the contrary, DPPH radical could not be quenched by SH at all [32]. The same researchers revealed superoxide anion radical quenching by SH and its activity is larger than P-334 but smaller than asterina-330.

The effect of SH on inflammation and skin aging of HaCaT cells was investigated [32]. The effect of SH on the mRNA levels of COX2, PCOLCE, and elastin are similar to those of P-334, although the effect on involucrin, which contributes to the formation of a cell envelope, is different from that of P-334. The involucrin mRNA level, which was increased by UV-irradiation, was suppressed dose-dependently by the addition of SH. The addition of 150 µM SH can keep the level at the basal UV-untreated condition. The protective effect was investigated of SH, isolated from *Scallop ovaries*, on human lung fibroblast (WI-38) cells from UV-induced cell death [34]. SH protects cells from UV-induced cell death in a concentration-dependent manner and the EC_50_ value is 64 µM, which is much smaller than that of P-334 (294 µM). This means SH shows a stronger protective capacity; nevertheless, the absorption maximum and coefficient of SH (λ_MAX_ = 334 nm, ε = 43,900 M^−1^·cm^−1^) are almost same as those of P-334 (λ_MAX_ = 334 nm, ε = 42,300 M^−1^·cm^−1^). The mechanism of protection is proposed to occur via a UV-screening effect of SH, because the viability rate was reduced to control levels when the medium containing SH was changed to medium without SH prior to UV irradiation. Growth promoting activities of SH on normal human skin fibroblast TIG-114 cells was also studied. SH did not suppress cell growth, implying that SH is not toxic: the cell proliferation rate was increased in a strongly concentration-dependent manner, though its activity is comparable to that of M-Gly and much larger than that of P-334. This difference in proliferation activity may be a possible reason for the higher UV protection activities of SH relative to P-334.

### 4.5. Mycosporine-2-Glycine (Figure 4(3f))

Mycosporine-2-glycine (M-2Gly) was first reported by Shick *et al.* in 1992 [91]. M-2Gly was isolated by HPLC from the extract of coral reef holoturoids and identified from the ratio of peak areas at 313 nm and 340 nm, (*i.e.*, the absorbance ratio) and its retention time was confirmed by co-chromatography with a standard M-2Gly from the sea anemone *Anthopleura elegantissima*. Strangely, the details of this standard were unpublished at that time except for the absorption maximum at 331 nm. In a following report, an additional explanation was provided [35]. Fractionation and alkaline hydrolysis yielded only glycine and its molar yields were approximately twice those obtained from M-Gly. Further, the molecular weight of M-2Gly was determined by ESI-MS (MH^+^ = 303). Almost a decade later, 1D- and 2D-NMR assignment was performed [92], although the configuration at the C-5 position is still unknown.

### 4.6. Asterina-330 (Figure 4(3g))

Purification of 70% ethanol extract obtained from the starfish *Asterina pectinifera* by several rounds of chromatography gave asterina-330 (A-330) as a colorless syrup [93]. Its structure was determined by HRMS and ^1^H-NMR spectra to be an aminocyclohexene imine substructure having a glycine and glycinol at both ends. The stereochemical configuration at the C5 carbon was reported to be of *R*-form by Kamio *et al.*, although detailed experimental evidence was not shown [94]. This report may be in error, because the same authors also reported the structure of palythine to be of *R*-form. The absorption maximum is 330 nm in water with an extinction coefficient of 43,800 M^−1^·cm^−1^. Esterification of A-330 with diazomethane gave the same corresponding aromatized ester as that of other MAAs. Photodecomposition of A-330 in the presence of riboflavin was studied and enhanced photosensitized degradation was observed at a rate constant of 7.00 × 10^−3^ m^2^·kJ^−1^, which is in a similar range to those of other aminocyclohexene imine-type MAAs. This result might involve the reaction of A-330 with a singlet oxygen formed via riboflavin sensitization, though the details are unknown. The researchers noted that the major degradation pathway is type-I photooxidation and not involving a reaction with a singlet oxygen [61].

Two research groups studied the lipid peroxidation inhibition capacity of A-330. Dunlap *et al.* tested AAPH-induced phosphatidylcholine peroxidation and revealed A-330 to be oxidatively stable in this experimental condition [30,90]. In contrast, the lipid peroxidation inhibition study evaluated by β-carotene bleaching method gave the opposite result. De la Coba *et al.* extracted the MAA mixture containing A-330 (86%) and Pi (14%) from *Gelidium corneum* and used for the lipid peroxidation inhibition test [31]. The result revealed that the inhibition capacity of A-330 is much stronger than that of M-Gly and is slightly stronger than P-334 and SH. This discrepancy on the activity may be due to the various differences in the experimental methods and conditions. We think that mediation of β-carotene is the critical factor for lipid peroxidation inhibition activity of MAAs because MAA is water-soluble and differs from phospholipid. A similar relationship is observed for ascorbic acid and vitamin-E with regard to lipid peroxidation inhibition. In fact, the inhibition capacity of P-334 on lipid peroxidation was enhanced by the addition of α-tocopherol [64].

The water-soluble ABTS radical quenching capacity of A-330 (containing small amount of Pi) is moderate in a basic medium, though it is greatly low in an acidic one [31] (Table 1). The IC_50_ values of A-330 under both acidic and basic pH are smaller than those of M-Gly and larger than those of P-334 and SH, though these two MAAs are also aminocyclohexene imine-type MAA. The reason for this difference is unclear becaue the p*K*_a_ values of these MAAs are unknown.

Scavenging of superoxides generated by the pyrogallol oxidation system was studied and the scavenging capacity of A-330 (containing slight Pi) was found to be stronger than those of SH and P-334 [31].

### 4.7. Palythinol (Figure 4(3h))

Palythinol (Pinol) had been isolated from the extract of the zoanthid *Palythoa tuberculosa* as a yellow crystal (mp = 155–156 °C) by using several types of chromatography [78]. Its structure was determined using 1D-NMR and IR spectra. Its absorption maximum is 332 nm in water with the absorption coefficient of 43,500 M^−1^·cm^−1^. Optical rotation of Pinol is [α]_D_ –51.9° (*c* = 1.3 in H_2_O). No inhibitory effect of Pinol against lipid peroxidation was confirmed by Dunlap *et al.* [30].

### 4.8. Palythine (Figure 4(3i))

Palythine (Pi) was isolated from the aqueous ethanol extract of *Chondrus yondoi* [95] and *Palythoa tuberculosa* [78]. Repeated chromatography on the carbon column yielded white crystal (mp = 155–156 °C [95] and 142–145 °C [78]). Hydrolysis of Pi with 1% NaOH at 40 °C for 1 h gave glycine and aminocyclohexenone (mp = 216–217 °C, λ_max_ 293 nm (acidic), 297 nm (basic)), indicating that the cyclohexene imine-type MAA consisting of a glycine and a NH_2_ group at both ends. Pi is stable under acidic conditions. The structure was confirmed by 1D-NMR and IR measurements, which support the inner salt form. Its absorption maximum appears at 320 nm (ε = 35,500 [95] or 36,200 [78] M^−1^·cm^−1^) and optical rotation is [α]_D_ = –7.9° (*c* = 1.5, H_2_O). The absorption maximum of Pi is shifted toward the longer wavelength region than that of M-Gly, indicating stronger π-conjugation character for Pi because of the inner salt form. X-ray crystallographic analysis of the colorless single crystal of Pi (with trihydrate) obtained from aqueous solution revealed the absolute configuration at the C5-carbon to be of *S*-form [96], which is consistent with other MAAs. This study also exhibited the versatile pattern of hydrogen bonding (12 types) and formation of three-dimensional hydrogen bonded structure.

Photodegradation of Pi in oxygen-saturated solution under irradiation by a Xenon lamp (400 W·m^−2^) was studied and only 4% of degradation was observed after 24 h [61]. After irradiation with UV-B light (280–315 nm, 0.78 W·m^−2^) for one hour, Pi was only slightly degraded [97]. The photodegradation quantum yield of Pi is very low ((1.2 ± 0.2) × 10^−5^), which agrees well with the highly photostable character of Pi in air-saturated aqueous solution [98]. In laser flash photolysis of acetone with Pi, the transient spectrum due to the triplet-triplet absorption of Pi was observed. The lifetime of this triplet state is *ca.* 9 µs, which is shorter than those of other amino-cyclohexene imine-type MAAs, including those of P-334 (14 µs) and SH (11 µs). This result suggests that the geometric isomerization around the C–N bond might contribute to the rapid deactivation. Photoacoustic calorimetry revealed non-radiative decay kinetics for the deactivation of excited Pi similar to P-334 and SH. Heat treatment of aqueous solutions of Pi at 60 °C for one hour resulted in slight degradation of Pi [97]. The inhibition property of Pi for AAPH-initiated phosphatidylcholine peroxidation has been studied and a negligibly small amount of degradation of Pi was observed, indicating that Pi is stable to oxidation in this lipid peroxidation system [30]. DPPH radical quenching reactions were carried out on the partially purified MAA extracts consisting of Pi, a small amount of A-330, and an unknown MAA (M-312) was studied [97]. Radical quenching occurred in a concentration-dependent manner and might be due to the effect of Pi, which is abundant in this MAA extract, although the experiment using the fully purified Pi will be necessary. Photosensitized reactions of Pi have been studied [61]. A 50% loss of Pi was achieved after two hours of exposure in the presence of rose bengal, though 100% was achieved after only one hour exposure in the presence of riboflavin. The photodegradation rate constant of Pi in the presence of rose bengal is 0.12 × 10^−3^ m^2^·kJ^−1^, but it becomes much larger in the presence of riboflavin (3.17 × 10^−3^ m^2^·kJ^−1^). This result indicates that the major degradation pathway does not involve the reaction of Pi with singlet oxygen even though both rose bengal and riboflavin can act as singlet oxygen source under photo-irradiation. The authors in this study proposed that type-I photooxidation mechanism as the major degradation pathway; however, their results do not deny the capacity for singlet oxygen quenching. Rastogi *et al.* investigated the reaction of Pi with hydrogen peroxide, but a sufficiently strong reaction was not observed [97].

### 4.9. Palythine-Threonine (Figure 4(3j))

Palythine-threonine (PT) with an absorption maximum at 320 nm was first found in the extracts from *Pocillopora eydouxi* and *Stillopora pistillata* [77]. The same MAA was isolated from the hermatypic coral *Pocillopora capitata* and its structure was determined [99]. The absorption maximum at 320 nm indicates the aminocyclohexene imine chromophore consists of one amino acid substituent, similar to palythine. Alkaline hydrolysis yielded only threonine; thus, palythine-threonine was identified. LC/MS/MS and HRMS analysis support this structure, though the absolute configuration is unknown.

### 4.10. Palythine-Serine (Figure 4(3k))

Palythine-serine (PS) was first found in the extract of *Pocillopora eydouxi* and its structure was determined from the results of LC/MS and NMR spectra [100]. The absorption maximum appears at 320 nm with an absorption coefficient of 10,500 M^−1^·cm^−1^.

### 4.11. Euhalothece-362 (Figure 4(3l))

**Scheme 1:**
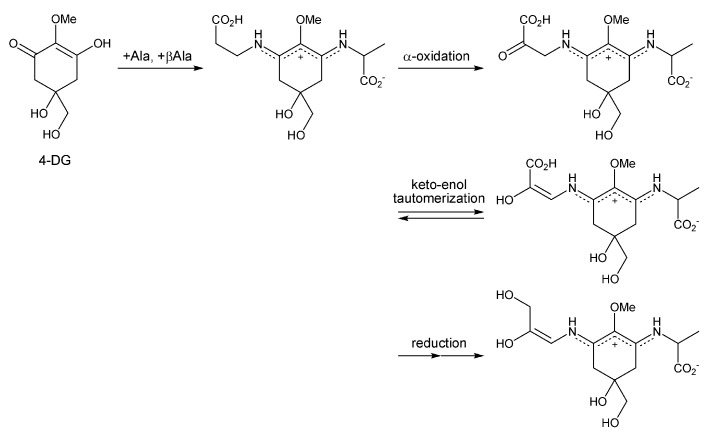
Plausible hypothesis for the biosynthetic pathway of Euhalothece-362.

Euhalothece-362 (E-362) was found in an HPLC chromatogram of the unicellular cyanobacterium *Euhalothece* LK-1 grown at high light intensities [92]. Its absorption maximum was 362 nm, similar to those of Pe and Ue, although their retention times were different. A further report proposed its structure based on the result of amino acid analysis of its hydrolysate and LC-MS/MS spectra [101]. The proposed structure is the two amino acids-substituted MAA consisting of alanine and 2,3-dihydroxypropenyl amine. The most red-shifted absorption band in all MAA molecules reported until now supports the presence of an additional conjugated double bond. Biosynthesis of this unique MAA has never been proposed. Based on the hypothesis that amino acid substitution on the MAA substructure (4-DG) is the first step of bio-transformation, 2,3-dihydroxypropenyl amine side chain is also thought to be derived from an amino acid. We developed a hypothesis of its plausible biosynthetic pathway from the retro-synthetic analysis (Scheme 1) of the side chain. The hydroxymethyl group can be obtained by reduction of the carboxylic acid, and the enole group can be in equilibrium with the ketone. Thus, β-alanine is the most plausible starting amino acid. Condensation of alanine and β-alanine with 4-DG gives mycosporine-alanine: β-alanine. A ketone group is made by the α-oxidation of the β-alanine side chain in equilibrium with the enol form. Because of the strong π-conjugation within the MAA substructure, the equilibrium of this tautomerization might be shifted toward enol-form. Reduction of the terminal carboxylic acid in thus formed enol form will give the objective structure. The biosynthetic pathway of this unique MAA will be clarified in the future.

### 4.12. Mycosporine-Glycine: Glutamic Acid (Figure 4(3m))

Mycosporine-glycine:glutamic acid (M-Gly:Glu) has been isolated (mp = 164–166°) and characterized from the extract of the sponge *Dysidea herbacea* [102]. Its structure was elucidated by amino acid analysis followed by alkaline hydrolysis for determining the amino acid components and MS, 1D- and 2D-NMR spectra were used for concluding the substitution pattern. The absorption maximum at 330 nm is consistent with the di-substituted aminocyclohexene imine-type MAA. The molar extinction coefficient of M-Gly:Glu is 43,900 M^−1^·cm^−1^, which is similar to that of SH (44,700).

### 4.13. Mycosporine-Glycine: Aspartic Acid (Figure 4(3n))

Mycosporine-glycine:aspartic acid (M-Gly:Asp) was first reported in 1985 [103]. The extract of the brine shrimp *Artemia* cysts was made using 80% ethanol and was purified using a Dowex column. Acid hydrolysis gave equimolar amounts of glycine and aspartic acid and an absorption maximum at 332–334 nm indicates the presence of an aminocyclohexene imine-type MAA with glycine and aspartic acid at both ends. Further reports on this compound remain to be published.

### 4.14. Mycosporine-Glycine: Valine (Figure 4(3o))

An MAA consisting of glycine and valine has been isolated from an extract of Antarctic marine organisms [104]. The chemical composition of the mycosporine-glycine:valine (M-Gly:Val) was determined by HPLC fractionation, followed by alkaline hydrolysis. The hydrolyzed amino acids were glycine and valine. The absorption maximum is 335 nm, although other chemical and physical properties have not been reported and there are no reports on the antioxidant activity of M-Gly:Val except regarding its sunscreening capacity.

### 4.15. Decarboxylated MAA Derivatives

Decarboxylation of the glycine chain in P-334 gives mycosporine-methylamine:threonine (Figure 4(3p), M-MA:Thr). This species was isolated from the reef-building corals *Pocillopora damicornis* and *Stylophora pistillata* [105], and the structure was identified by 1D- and 2D-NMR spectroscopy, high-resolution mass spectroscopy (HRMS), and amino acid analysis. The determined structure is an aminocyclohexene imine substructure bearing threonine and methylamine substituents; though, to the best of our knowledge, the absolute configuration has not been identified yet. The UV absorption spectrum of M-MA:Thr shows an absorption maximum at 330 nm with a molar extinction coefficient of 33,000 M^−1^·cm^−1^.

Decarboxylation of the glycine chain in SH yields mycosporine-methylamine:serine (Figure 4(3q), M-MA:Ser), which was isolated from *Pocillopora eydouxi* [100] and whose structure was identified by ^1^H-NMR and LC/ESI-MS spectra. The determined structure is an aminocyclohexene imine substructure bearing methylamine and serine substituents. The absolute configuration has not yet been identified. The UV absorption spectrum of M-MA:Ser shown an absorption maximum at 325 nm with a molar extinction coefficient of 16,600 M^−1^·cm^−1^.

Recent studies of the novel MAAs extracted from the sea hare *Aplysia californica* revealed the aminocyclohexene imine-type MAAs consisting of glycine and several decarboxylated amino acids at both ends, such as threonine, alanine, and glycine [94]. The chemical structures were determined by NMR, MS and absorption spectra, and they were named aplysiapalythine A (APA, Figure 4(3r)), B (APB, Figure 4(3s)), and C (APC, Figure 4(3t)). The researchers proposed the absolute configuration of these MAA substructures to be of the *R*-form, based on the NMR method described in a previous report [56]; though, more detailed explanation is still required. Considering the fact that APA has a decarboxylated P-334 structure, it is reasonable to expect that APA is synthesized by a similar biosynthetic pathway as P-334 and the configuration at the C-5 carbon in APA might be inherited from P-334. Notably, the stereogenic center can be changed from *S* to *R*, because the priority number of substituents can be changed due to decarboxylation. They also determined the configuration of the known MAAs palythine (Pi) and asterina-330 (A-330) to be of the *R*-form, although the known configuration of Pi revealed by X-ray crystallographic analysis is *S*-form [96]. Stereochemical assignment of these two MAAs might be an error. The absorption maxima of APA, APB, and APC are 332, 332, and 320 nm, respectively.

### 4.16. Hybrid MAAs (MAAs Consisting of Multiple Chromophores)

Few examples exist of hybrid MAAs comprising several MAA chromophores in one molecule. Carreto *et al.* reported three types of hybrid MAAs isolated from the extracts of the surface bloom-forming toxic dinoflagellates *Alexandrium tamarense*, *Alexandrium catenella*, and *Alexandrium minutum* [86].

One of these hybrid MAAs shows an asymmetric absorption spectrum with a maximum at 320 nm that is thus named M-320. The spectrum is similar to the combined spectra of SH and M-Gly, indicating two types of MAAs. Acid hydrolysis yielded these MAAs in a molar ratio close to one. The mass spectrum of M-320 exhibited a peak at *m/z* = 558.5 (in negative mode) and at *m/z* = 560.6 (in positive mode); thus, a molecular weight of M-320 (559.5 Da) is expected, which is 18 units lower than the calculated net mass of SH (332.3 Da) and M-Gly (245.2 Da). These results indicate this compound is the condensate of SH and M-Gly. Based on the structure of these two molecules, the possible linkage is thought to be formed by an ester bond; however, the bonding scheme has not yet been reported. This prediction is supported by the study of MAA biosynthesis induced by the photosynthetically active radiation (PAR) in *Alexandrium tamarense* [87].

Another MAA shows the absorption maximum at 335 nm with a shoulder peak at 360 nm, indicating two distinct MAA chromophores. Thus, this hybrid MAA was temporarily named M-335/360. Acid hydrolysis yielded SH, Pe, and Pi in a molar ratio of 1.0:0.6:0.4. Considering the unstable nature of Pe during hydrolysis, it has been suggested that M-335/360 contains SH and Pe in a 1:1 molar ratio. The UV-visible absorption spectrum of M-335/360 can be reconstructed from the spectra of these MAAs. The negative-ion mass spectrum for M-335/360 showed a peak at an *m/z* of 597.6, indicating that the expected molecular weight is 598.6 Da. This is 18 units lower than the calculated mass derived from the summation of SH (332.3 Da) and Pe (284.1 Da). These results support the hybrid MAA consisting of SH and Pe formed by condensation and most likely linked via an ester bond. This hypothesis is also strengthened by the result of MAA biosynthesis in *Alexandrium tamarense* under the PAR [87].

M-328/360 is the other MAA with an absorption maximum at 328 nm, which has an extended plateau and a clear shoulder peak at 360 nm. Acid hydrolysis of this hybrid MAA yielded SH, M-Gly, and Pe as products in a molar ratio of 1.0:1.0:0.6, respectively. Pi and small amounts of unknown species with maximum absorption values at 320 nm were also detected as secondary products. Therefore, M-328/360 is thought to consist of SH, M-Gly and Pe in a 1:1:1 molar ratio, though no results currently exist to explain bonding scheme.

## 5. Derivatized MAAs (Figure 5)

**Figure 5:**
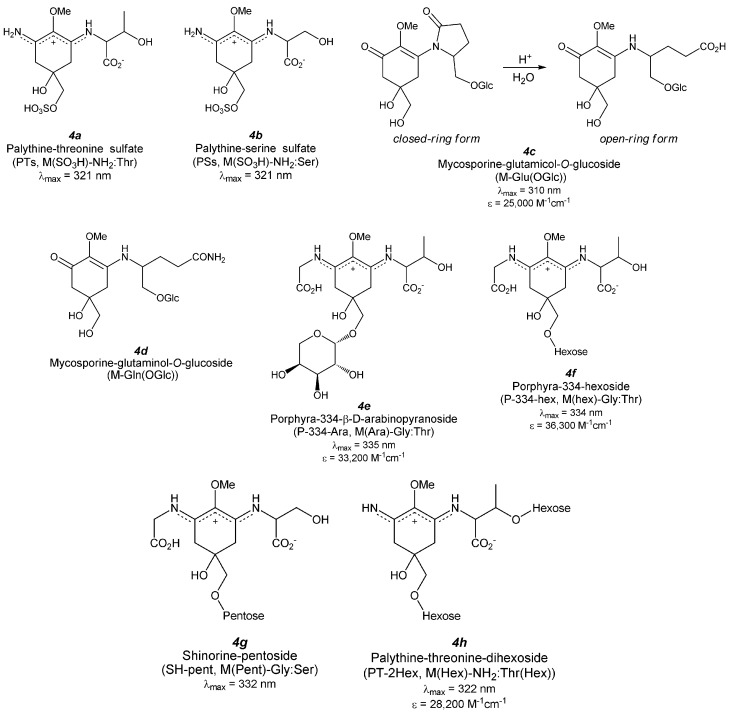

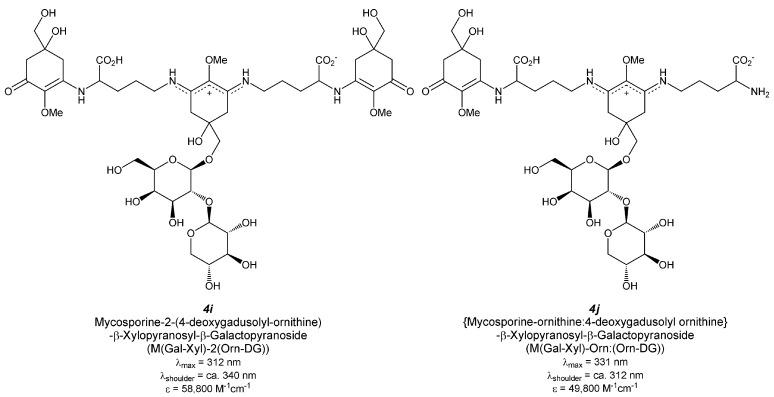
Chemical structures and spectroscopic properties of derivatized MAAs. Abbreviations are in parenthesis.

### 5.1. MAA Sulfates

MAA sulfates are found in only in one specific coral and no diversity of these derivatives in other organisms has been reported until now. In this section, the structure determination of two sulfates are mentioned briefly.

#### 5.1.1. Palythine-Threonine Sulfate (Figure 5(4a))

HPLC analysis of the methanol extract of a reef-building coral *Stylophora pistillata* revealed the existence of the highly polar MAA. Won *et al.* isolated this substance and determined the structure by HRMS and 1D-NMR analysis [106]. HRMS analysis (*m/z* 367.08201) suggested a formula of C_12_H_19_N_2_O_9_S and NMR data showed good agreement with M-MA:Thr. The absorption maximum at 321 nm indicates the presence of a chromophore with an unsubstituted imine group and a substituted amino acid on the MAA substructure. A downfield shift of C7 (hydroxymethyl group) indicates a substitution that increases the polar nature of this MAA. These spectral data support the structure of palythine-threonine sulfate (PTs), although the stereochemical configuration has not been clarified yet.

#### 5.1.2. Palythine-Serine Sulfate (Figure 5(4b))

The sulfate ester of palythine-serine (PSs) had been found in same coral that contains the other sulfate ester, PTs [106]. The PSs structure was determined in a similar way to that of PTs. The absorption maximum appears at 321 nm, which is same as that of PTs. Other properties of this compound have not yet been elucidated.

### 5.2. MAA Glycosides

MAA glycosides have been found in several organisms, mainly in terrestrial species of fungi, fresh water yeasts of basidiomycetous affiliation, microcolonial ascomycetes, and cyanobacteria. The former three organisms produce aminocyclohexenone-type MAAs including glutaminol and/or glutamicol-substituted MAAs with glucose substitution. The latter organism produces various types of aminocyclohexene imine-type MAAs.

#### 5.2.1. Mono-Substituted MAA Glycosides (Aminocyclohexenone-Type MAA Glycosides)

##### 5.2.1.1. Mycosporine-Glutamicol-O-Glucoside (Figure 5(4c))

Glycosylated MAA comprising M-Glu(OH) has been found in the three fungal plant *Ascochyta pisi*, *Cladosporium herbarum*, and *Septoria nodurum,* and its structure was determined by MS spectroscopy [48]. The HPLC chromatogram revealed that this compound dominantly exists in a closed-ring form (95%–100%), and acid hydrolysis showed that it first converts into an open-ring form and then into the aglycone as an open-form, and concomitantly glucose. The researchers proposed the substitution position of glucose on the CH_2_OH group at C9 carbon position based on the fragmentation pattern of M-Glu(OH). Careful MS/MS analysis of rock-inhabiting microcolonial fungi demonstrated the final conclusion of this novel glucosylated mycosporine-like compound (M-Glu(OGlu)) [107,108]. The absorption maximum and molar absorption coefficient of M-Glu(OGlc) are 310 nm and 25,000 M^−1^cm^−1^ in the closed-ring form, respectively.

##### 5.2.1.2. Mycosporine-Glutaminol-O-Glucoside (Figure 5(4d))

Glycosylated MAA comprising M-Gln(OH) has been found in several terrestrial fungi including *Pyronema omphalodes* [50] and *Trichothecium roseum* [51]. Acidic hydrolysis (0.5 N HCl, 100 °C) yielded at first M-Gln(OH) and glucose and then M-Glc(OH), indicating the structure as mycosporine-glutaminol glucoside (M-Gln(OGlu)). The structure was finally determined by ^1^H- and ^13^C-NMR and MS spectra. NMR results revealed the glucosidic linkage occurs at the glutaminol chain via a glycosidic bond with a configuration of α (*J* = 3.44 Hz).

#### 5.2.2. Di-Substituted MAA Glycosides (Aminocyclohexene Imine-Type MAA Glycosides)

##### 5.2.2.1. Porphyra-334 Glycosides (Figure 5(4e,f))

Arabinose-bound P-334 (P-334-Ara, M(Ara)-Gly:Thr) was isolated from the extract of the genotype A of *Nostoc commune* [39,67] as the major MAA. MALDI-TOF MS/MS analysis was performed on the molecular ion fragment and gave a fragment with an *m/z* of 347 as a second mass, which is identical to that of P-334. The neutral loss of 132 suggests the presence of a pentose as a substituent. The IR spectrum shows similar absorption peaks of P-334 and the strongest absorption peak is assignable to hydroxyl groups. The absorption maximum at 335 nm with an absorption coefficient of 33,200 M^−1^cm^−1^ is comparable to that of P-334 (334 nm, 42,300 M^−1^cm^−1^). Acid hydrolysis followed by acetylation showed penta-*O*-acetylarabinol (arabinose derivative) in the GC-MS chart, indicating the presence of an arabinose moiety. The substructure was confirmed by 1D- and 2D-NMR analyses. HMBC correlation spectrum revealed the arabinose substitution at the C7 carbon position via an *O*-glycoside bond. The large coupling constant at the anomeric proton indicates the arabinose is present in the β-form. The magnetically anisotropic methylene proton present in the arabinose moiety indicates a pyranose-form of arabinose. The ABTS radical scavenging capacity of this arabinose substituted P-334 derivative was evaluated by monitoring the ESR signal reduction and it was found that antioxidant property was as strong as that of Trolox.

The hexose-substituted P-334 (P-334-hex, M(hex)-Gly:Thr) was isolated from the extract of the genotype D of *Nostoc commune* [69]. The absorption maximum at 334 nm with an extinction coefficient of 36,300 M^−1^·cm^−1^ suggests an aminocyclohexene imine-type chromophore. MALDI-TOF MS/MS analysis performed on the molecular ion fragment gives a fragment peak identical to P-334; thus, the neutral loss of 162 Da indicates the presence of hexose as a substituent. The IR spectrum shows similar absorption peaks as P-334 and the strong absorption peak of hydroxyl groups. 1D- and 2D-NMR analyses also suggest hexose-substituted P-334 and the substitution position is C7 methylene in P-334 backbone via an *O*-glycoside linkage. Comparing the coupling constant at the anomeric proton and the chemical shifts for all carbon and proton atoms with those of methyl-α-d-mannose suggests the substitution of α-d-mannose. Another hexose-bound P-334 had also been found in genotype A of *Nostoc commune* [39] as very minor MAAs. The absorption maximum at 334 nm supports an aminocyclohexene imine substructure. The neutral loss of 162 Da from the parent molecular ion fragment in the MALDI-TOF MS/MS analysis predicts hexose-substituted P-334 as a possible candidate. A type of hexose and substitution position has not been determined; however, substitution does not occur at the C7 methylene carbon in the P-334 substructure because the retention time of this compound is different from that of the previously reported hexose-bound P-334. ABTS radical scavenging by C7-hexose-substituted P-334 was tested by the ESR method. Interestingly, the scavenging capacity is weaker than that of Trolox, though a pentose-bound analogue showed a similar scavenging capacity to that of Trolox.

##### 5.2.2.2. Shinorine Glycoside (Figure 5(4g))

Pentose-substituted shinorine (SH-pent, M(pent)-Gly:Ser) was recently found in genotype A of *Nostoc commune* [39] as a minor MAA. The absorption maximum at 332 nm indicates an aminocyclohexene imine substructure. MALDI-TOF MS/MS analysis on the parent molecular ion fragment gives a fragment with an *m/z* of 333, which is identical to the molecular mass of SH. The neutral loss of 132 Da suggests the deletion of a pentose, predicting a pentose-substituted SH as a possible candidate. NMR chemical shifts support the existence of an SH substructure and further the lower-shifted signal indicates that pentose substitution occurs at C7 via an *O*-glycoside bond.

##### 5.2.2.3. Palythine-Threonine Glycoside (Figure 5(4h))

Two hexose-bound palythine-threonine (PT-2Hex, M(hex)-NH_2_:Thr(hex)) MAAs were identified from the extract of the *Nostoc commune* genotype D [69]. Its absorption maximum appears at 322 nm, which is consistent with PT (320 nm). Garcia-Pichel and Castenholz noted novel MAAs with absorption maxima at 324 to 326 nm; the palythine-threonine derivative may correspond to one of those described in 1993 [109]. The HRMS spectrum reveals an *m/z* value of 613.2462, indicating a molecular formula of C_24_H_41_N_2_O_16_. MALDI-TOF MS/MS analysis of the parent molecular ion peak shows the two different neutral losses of 162 Da, indicating the presence of two hexose substituents. The presence of hexose is also supported by the IR spectrum. 1D- and 2D-NMR spectra revealed the palythine-threonine (PT) substructure and the substitution positions of two hexoses at the C7 carbon in the PT backbone and at the threonine-chain via *O*-glycoside bonds. The absolute configuration of this new MAA-glycoside has not been elucidated. The molar absorption coefficient is 28,200 M^−1^ cm^−1^.

Another MAA with an absorption maximum (PT-Hex) at 322 nm was purified from the same *Nostoc commune* colonies. The absorption maximum and a molecular mass of 450 Da were determined by MALDI-TOF MS and FAB MS spectra, indicating the presence of a hexose and a PT scaffold. MS/MS analysis suggests the hexose is not bound to the threonine chain, and thus PT-Hex can be expected to have a PT substructure with a hexose substituent at the C7 carbon. This prediction indicates PT-Hex can be bio-synthesized as a precursor of PT-2Hex.

The ABTS radical scavenging of PT-2Hex was also investigated and was found to be stronger than that of P-334-hex and weaker than those of ascorbic acid and Trolox. This relatively weaker reactivity with radical species is similar to that of other aminocyclohexene imine-type MAAs.

##### 5.2.2.4. Hybrid MAA Glycoside (Figure 5(4i,j))

A characteristic MAA with double absorption maxima at 312 and 340 nm isolated from a terrestrial cyanobacterium was reported by Scherer *et al.* [110]. The atypical MAA was also described by Garcia-Pichel and Castenholz [109], who thought that the MAA contains two typical MAA chromophores: the aminocyclohexanone-type with an absorption maximum at 310 nm and the aminocyclohexene imine-type with an absorption maximum at 340 nm. This is a logical proposal based on the characteristic absorption spectrum, which was so unique that it remained to be elucidated until 20 years later when we determined the chemical structure of the 1050-Da MAA (Figure 5(4i)), which represents the largest MAA that has thus far been reported [39,67].

In 1995 Böhm *et al.* reported an oligosaccharide-bound MAA from *Nostoc commune* [111]. The proposed hypothetical structure was unique and its molecular mass was much larger than those of typical MAAs. However, the hypothetical structure could not explain the double absorption maxima in the unique compound, although Böhm *et al.* [111] mentioned that “every peak in the HPLC chromatogram absorbs at 312 and 335 nm”. There are three reasons for the limited structural analysis by Böhm *et al.* at that time. First, the extracts from *Nostoc commune* contain a significant amount of polysaccharides from the extracellular matrix that may be fragmented during the MAA extraction and purification steps. Moreover, these oligosaccharides are poorly detected by UV absorption because the elution of MAAs is normally monitored at 330 nm, a wavelength that does not enable detection of sugars. Thus, the purified MAA from *Nostoc commune* may contain oligosaccharides as an impurity, which could mislead the explanation of the data from the structural analysis. We almost made a similar error in response to the MS spectrum, which suggests the presence of oligosaccharides in our shinorine-derivative sample from *Nostoc commune* [39,112]. Notably, the MS spectrum published by Böhm *et al.* [111] contained molecular ion fragments with *m/z* values of 1050 and 881 overlapping with molecular ion fragments from oligosaccharides. The fragment with an *m/z* value of 1051 can be attributed to the parental ion of the 1050-Da MAA (Figure 5(4i)) and the fragment with the *m/z* value of 881 corresponds to the partially degraded ion (Figure 5(4j)) derived from the 1050-Da MAA. These peaks were elucidated in our studies later [39,67]. Second, MAAs from *Nostoc commune* are glycosylated [39,67,69]. The glycosylated MAAs are likely unique to terrestrial habitats [107,108]. Thus far, glycosylated MAAs are atypical MAAs that have only been reported in limited numbers of organisms; *Nostoc commune* is a typical and the most intensively studied example. The structural analysis of MAA glycosides is often complex when the contamination of oligosaccharides is not directly noted. Third, there is genetic polymorphism in *Nostoc commune* [113] and this is related to the diversity of the MAA types in this organism [39,67,69,112]. *Nostoc commune* is considered one species and the types are nearly indistinguishable morphologically. When these biologically different origins are mixed together and then used for the characterization of MAAs, the results become complicated. This may be one reason explaining the HPLC chromatogram by Böhm *et al.* [111], in which at least 7 peaks were detected.

Because the genetic differences in *Nostoc commune* genotypes were revealed [113], we had a great advantage through which to characterize the chemical diversity of MAAs in this species [39,67,69,112]. The technical innovations in the MS analysis in the past decade helped us to solve the unique structure of the 1050-Da MAA glycoside in *Nostoc commune*. An MAA with absorption maxima at 312 and 340 nm was found in genotype B *Nostoc commune* as a major MAA component and its structure was determined [39,67]. The presence of the chromophores aminocyclohexenone and aminocyclohexene imine was directly demonstrated by the analysis of thermally decomposed products on LC-MS; fragments with single absorption peaks at 312 nm and at 335 nm were identified, which correspond to the respective chromophores [67]. The sugar composition was determined by GC-MS analysis of the acetylated hydrolysate of MAA, and hexa-*O*-acetylgalactitol (galactose derivative) and penta-*O*-acetylxylitol (xylose derivative) were detected, indicating the presence of galactose and xylose moieties [39]. Their coupling scheme was determined by the MS/MS pattern and detailed NMR analysis (Figure 5(4i)). The absorption coefficient at 312 nm is 58,800 M^−1^·cm^−1^, which is the largest coefficient of all reported MAAs and is due to the multiple chromophores.

Another MAA with absorption bands at 331 and 312 nm (as a shoulder) was also isolated from same *Nostoc commune* colony. The molecular mass of 880 Da suggests the removal of the 4-deoxygadusol unit from the 1050-Da hybrid MAA. 1D- and 2D-NMR, MALDI-TOF MS/MS, and IR spectra analyses support this prediction (Figure 5(4j)). The absorption coefficient at 331 nm was 49,800 M^−1^·cm^−1^.

The terrestrial cyanobacterium *Nostoc commune* is known as an anhydrobiotic microorganism with oxygenic photosynthetic capabilities without differentiation into akinetes (spores) [114]. *Nostoc commune* has no metabolic activity in desiccated states, though the species is able to rapidly resume metabolism upon rehydration [115,116]. This phenomenon is termed “anhydrobiosis” [117,118]. The mechanism of extreme desiccation tolerance by this species is thought to involve multiple processes that include extracellular polysaccharide (EPS) production, compatible solute accumulation, and regulation of photosynthesis to protect cells from damage during desiccation. Moreover, desiccated *Nostoc commune* can retain viability for over 100 years following desiccation [119,120], implying the involvement of potent antioxidants protecting biomolecules from oxidation, which must be chemically stable and active over a long term. Hence, we characterized low-molecular-weight antioxidants in *Nostoc commune*. In our study, the radical scavenging activity related to MAAs was found in water extracts [67]. It has been reported that porphyra-334 plays a photoprotective role but does not function as a strong antioxidant [30,60,62]. The 478-Da MAA from *Nostoc commune*, the arabinose-bound porphyra-334 derivative, showed ABTS radical scavenging activity [67]. The aminocyclohexanone-type MAAs show radical scavenging activity *in vitro* according to their chemical structures, although the aminocyclohexene imine-type MAAs are less active in general. This result suggests the possible role of the imine-type MAA as a stock for the strong antioxidants carbonyl-type MAAs, although this hypothesis requires further evaluation. A unique characteristic of *Nostoc commune* might be that glycosylation leads to an antioxidant role for porphyra-334, indicating the advantage of glycosylation to MAAs. Moreover, *Nostoc commune* has the hybrid-type of MAA glycoside with both the aminocyclohexenone and aminocyclohexene imine chromophores, and its radical scavenging activity is stronger than that of the arabinose-bound porphyra-334 derivative [67]. This enhancement of scavenging activity suggests a possible reason why *Nostoc commune* of genotype B has acquired the hybrid-type MAAs. The biosynthetic pathway of these hybrid MAA glycosides are not fully understood, though it is reasonable to think that the glycosylated imine-type MAA might be coupled with two carbonyl-type MAAs because of the glycosylation position located only at the central MAA chromophore. Studying the reaction mode of radical scavenging and identifying the relevant genes will enable us to clarify the involvement of MAAs in anhydrobiosis.

The multiple roles that MAAs and their derivatives, particularly those of the carbonyl-type, play as sunscreens against UV radiation and as radical scavengers against oxidative stresses have been well established [121,122]. The induction of MAAs in response to thermal and saline stresses suggest additional protective functions against environmental stress [123], although there is a skeptical view on the role as compatible solutes because the contribution of MAA to the cellular osmotic pressure may be overestimated [121]. The physiological functions of MAAs *in vivo* remain to be directly demonstrated. Recently the genes involved in MAA biosynthesis have identified in cyanobacteria and the loss-of-function and gain-of-function analysis using these genes will help answer the remaining questions of how MAAs function as stress protectants.

## 6. Conclusions

In this review, we surveyed all the reported MAA derivatives, including their glycosides found in the chemical abstracts service (CAS) database and summarized their biological sources, manner of structure determination, chemical- and physical-properties, antioxidant activity (*in vitro*), and physiological properties. Recent studies have revealed their radical scavenging activities *in vitro*; the role of MAA species *in vivo* is not fully elucidated. The antioxidant crosstalk between *in vivo* antioxidants including MAAs is prospective, although it remains to be studied in the future. Modifications (at least glycosylation) of MAA molecules and hybridization with multiple chromophores could enhance their antioxidant activities, but the relevance of the derivatized MAAs to the functional roles of these molecules remains to be revealed. Further, a comprehensive understanding of the chemical diversity of MAAs and their biological functions will reveal the strategy used in living organisms to adapt to UV radiation and associated oxidative effects. The multi-functions of MAAs from diverse biological sources may have commercial applications, e.g., cosmetics, dietary supplements, medicine, functional organic devices and others. Namely, water-soluble MAA derivatives can become candidates for such cosmetic applications because these compounds have high photo-resistance and antioxidant activities. In this context, the survey of new MAA derivatives, especially those bearing carbohydrates, is a promising endeavor. In addition, development of MAA synthetic pathway is also necessary for the further application of these compounds in the nutritional fields, including dietary supplements.

## Appendix

**Table A1:** Summary of the antioxidant activities of various MAA derivatives.

Compound Name	Antioxidant Activity							Physiological Property
	ORAC	Lipid Peroxidation Inhibition	Radical Quenching Capacity			Singlet Oxygen Quenching	Hydrogen Peroxide Quenching	Protecting Effect on Living Cell
			DPPH	ABTS	Superoxide			
*MAA precursors*								
Gadusol Figure 2(1a)	yes [21] *^a^*	–	–	yes [21] *^a^*	–	–	–	–
4-Deoxygadusol Figure 2(1b)	–	yes [26]	–	–	–	yes [27] *^b^*	–	–
*Aminocyclohexenone-type MAAs*								
Mycosporine-glycine Figure 3(2a)	–	yes [30] *^c^* yes [31] *^d^*	yes [32] *^e^*	yes [31] *^d^*	no [31] *^d^*	yes [30] *^c^*	–	yes [34] *^f^*
Mycosporine-GABA Figure 3(2e)	–	–	–	yes [39] *^g^*	–	–	–	–
*Aminocyclohexene imine-type MAAs*								
Porphyra-334 Figure 4(3a)	–	no [30] *^h^* yes [31] *^i^* yes [64] *^j^*	weak [32] *^e^* weak [65] *^j^*	weak [31] *^i^*	moderate [31] *^i^*	no [61] yes [64] *^j^*	–	yes [31] *^i^* yes [32] *^e^* yes [72] yes [74] *^j^* yes [75] *^j^*
dehydrated porphyra-334	–	–	yes [65] *^j^*	–	–	–	–	–
Palythenic acid Figure 4(3b)	–	–	–	–	–	no [61]	–	–
Palythene, Usujirene Figure 4(3c, 3d)	–	yes [82] *^j^*	–	–	–	no [61]	–	–
Shinorine Figure 4(3e)	–	no [30] *^k^* yes [31] *^l^*	no [32] *^e^*	weak [31] *^l^*	moderate [31] *^l^*	no [61]	–	–
Asterina-330 Figure 4(3g)	–	no [30] *^m^* yes [31] *^n^*	–	yes [31] *^n^*	yes [31] *^n^*	no [61]	yes [97] *^o^*	–
Palythinol Figure 4(3h)	–	no [30] *^c^*	–	–	–	–	–	–
Palythine Figure 4(3i)	–	no [30] *^c^*	yes [97] °	–	–	very weak [61]	weak [97] *^o^*	–
*Glycosylated MAAs*								
P-334 arabinoside Figure 5(4e)	–	–	no [67] *^g^*	yes [67] *^g^*	–	–	–	–
P-334 hexoside Figure 5(4f)	–	–	–	yes [69] *^g^*	–	–	–	–
Palythine-threonine-dihexoside Figure 5(4h)	–	–	–	yes [69] *^g^*	–	–	–	–
Hybrid MAA-1 (1050Da) Figure 5(4i)	–	–	yes [67] *^g^*	yes [67] *^g^*	–	–	–	–
Hybrid MAA-2 (880Da) Figure 5(4j)	–	–	–	yes [39] *^g^*	–	–	–	–

– not tested. MAAs are typically purified from the alcoholic water extracts of living organisms, for example *^a^* fish roes, *^b^* phytoplankton (*P. micans*), *^c^* zoanthid (*P. tuberculosa*), *^d^* lichen (*L. pygmaea*), *^e^* marine green algae (*C. hedleyi*), *^f^* scallop (*P. yessoensis*), *^g^* terrestrial cyanobacteria (*N. commune*), *^h^* red algae (*P. tenera*), *^i^* red algae (*P. rosengurttii*), *^j^* laver (*P. yezoensis*), *^k^* ascidian (*L. patella*), *^l^* red algae (*A. devoniensis*), *^m^* coral trout (*P. leopardus*) lens, *^n^* red algae (*G. corneum*) and *^o^* cyanobacteria (*Lyngbya* sp.).

**Table A2:** Summary of the references to determine the chemical structure of each MAA.

Compound Name	References
*MAA precursors*	
Gadusol Figure 2(1a)	[17,18]
4-Deoxygadusol Figure 2(1b)	[24]
*Aminocyclohexenone-type MAAs*	
Mycosporine-glycine Figure 3(2a)	[24,29]
Mycosporine-taurine Figure 3(2b)	[35]
Mycosporine-alanine Figure 3(2c)	[37]
Mycosporine-β-alanine Figure 3(2d)	[38]
Mycosporine-GABA Figure 3(2e)	[39]
Mycosporine-serine Figure 3(2f)	[40]
Mycosporine-serinol Figure 3(2g)	[43,44]
Mycosporine-glutamic acid Figure 3(2h)	[46]
Mycosporine-glutamicol Figure 3(2i)	[41,47]
Mycosporine-hydroxyglutamicol Figure 3(2j)	[49]
Mycosporine-glutamine Figure 3(2k)	[50]
Mycosporine-glutaminol Figure 3(2l)	[51,52]
*Aminocyclohexene imine-type MAAs*	
Porphyra-334 Figure 4(3a)	[53]
Palythenic acid Figure 4(3b)	[76]
Usujirene Figure 4(3c)	[80]
Palythene Figure 4(3d)	[78,79]
Shinorine Figure 4(3e)	[83,84]
Mycosporine-2-glycine Figure 4(3f)	[91]
Asterina-330 Figure 4(3g)	[93]
Palythinol Figure 4(3h)	[78]
Palythine Figure 4(3i)	[78,95]
Palythine-threonine Figure 4(3j)	[77,99]
Palythine-serine Figure 4(3k)	[100]
Euhalothece-362 Figure 4(3l)	[101]
Mycosporine-glycine:glutamic acid Figure 4(3m)	[102]
Mycosporine-glycine:aspartic acid Figure 4(3n)	[103]
Mycosporine-glycine:valine Figure 4(3o)	[104]
Mycosporine-methylamine:threonine Figure 4(3p)	[105]
Mycosporine-methylamine:serine Figure 4(3q)	[100]
Aplysiapalythine A Figure 4(3r), B Figure 4(3s), and C Figure 4(3t)	[94]
*Derivatized MAAs*	
Palythine-threonine sulfate Figure 5(4a)	[106]
Palythine-serine sulfate Figure 5(4b)	[106]
Mycosporine-glutamicol-*O*-glucoside Figure 5(4c)	[48]
Mycosporine-glutaminol-*O*-glucoside Figure 5(4d)	[50,51]
Porphyra-334-β-d-arabinopyranoside Figure 5(4e)	[39,67]
Porphyra-334-hexoside Figure 5(Figure 5)	[69]
Shinorine-pentoside Figure 5(4g)	[39]
Palythine-threonine-dihexoside Figure 5(4h)	[69]
Mycosporine-2-(4-deoxygadusolyl-ornithine)-β-xylopyranosyl-β-galactopyranoside Figure 5(4i)	[39,67]
(Mycosporine-ornithine:4-deoxygadusolyl ornithine)-β-xylopyranosyl-β-galactopyranoside Figure 5(4j)	[39]
